# The Application of Auto-Disturbance Rejection Control Optimized by Least Squares Support Vector Machines Method and Time-Frequency Representation in Voltage Source Converter-High Voltage Direct Current System

**DOI:** 10.1371/journal.pone.0130135

**Published:** 2015-06-22

**Authors:** Ying-Pei Liu, Hai-Ping Liang, Zhong-Ke Gao

**Affiliations:** 1 School of Electrical and Electronic Engineering, North China Electric Power University, Baoding, Hebei Province, 071003, China; 2 School of Electrical Engineering and Automation, Tianjin University, Tianjin, 300072, China; University of California Berkeley, UNITED STATES

## Abstract

In order to improve the performance of voltage source converter-high voltage direct current (VSC-HVDC) system, we propose an improved auto-disturbance rejection control (ADRC) method based on least squares support vector machines (LSSVM) in the rectifier side. Firstly, we deduce the high frequency transient mathematical model of VSC-HVDC system. Then we investigate the ADRC and LSSVM principles. We ignore the tracking differentiator in the ADRC controller aiming to improve the system dynamic response speed. On this basis, we derive the mathematical model of ADRC controller optimized by LSSVM for direct current voltage loop. Finally we carry out simulations to verify the feasibility and effectiveness of our proposed control method. In addition, we employ the time-frequency representation methods, i.e., Wigner-Ville distribution (WVD) and adaptive optimal kernel (AOK) time-frequency representation, to demonstrate our proposed method performs better than the traditional method from the perspective of energy distribution in time and frequency plane.

## Introduction

In recent years, owing to the depletion of fossil fuels and the serious environmental pollution caused by fossil fuels burning, the development and utilization of renewable energy is of great significance throughout the world [1–3]. Consequently, the technologies such as renewable energy generation and grid connection technology, related energy storage, grid-vehicle interaction draw a great deal of attentions from different research fields. As one of the most mature renewable resources, wind farms integration into the electric power grid has been well investigated [4–5]. In view of the fact that the connection between wind farms and the grid usually covers far distance, the multi-terminal direct current transmission is particularly preferable for wind power transmission [6–7]. In regard to energy storage, three different electrochemical energy storage systems are comparatively examined for a hybrid bus powertrain operated in Gothenburg, Sweden [8]. Various types of energy storage have been discussed including compressed air energy storage, flywheel energy storage, battery energy storage, super capacitor energy storage [9]. As one of the research hotspots, the grid-vehicle interaction has been investigated under the renewable energy background [10–11]. Saber et al. investigated the cost and emission reduction in a smart grid in terms of maximum utilization of gridable vehicles and renewable energy resources [12].

In China, the energy resources focus on the western region, but the electricity power demand in middle and eastern region greatly increase [13–14]. In order to guarantee the adequate supply of electricity power in the middle and eastern area, some solutions have to be implemented to transmit electricity power to the load center. High voltage direct current (HVDC) transmission can realize power transmission with long distance and large capacity. In the traditional HVDC system, the converter consists of thyristors, which led to some disadvantages, e.g., the power network connected by HVDC must be an active network; the reactive power consuming is high when it runs, so there must be many reactive power compensators in the HVDC system.

In order to solve the above problems fundamentally, the full-controlled device should be used instead of thyristor. The voltage source converter-high voltage direct current (VSC-HVDC) transmission is a new generation of HVDC, and the VSC-HVDC is based on full-controlled device such as IGBT or GTO, voltage source converter and new pulse modulation technique. Compared to the traditional HVDC, it has many advantages, e.g., it can supply electricity power to the load directly; the active power and the reactive power can be controlled independently and flexibly.

The investigations on VSC-HVDC mainly focus on the mathematical modelling, control strategy, multi-terminal HVDC systems, flow control, the transient voltage stability, unbalanced operation, etc. Refs. [15–16] present the control strategy for VSC-HVDC system. The flow control in VSC-HVDC system has been studied in Refs. [17–18]. The mathematical models of VSC-HVDC refer to Refs. [19–20]. The protection strategy of VSC-HVDC system has been investigated in Refs. [21–22]. Yuan et al. [23] studied the unbalanced three-phase control strategy of VSC-HVDC system. Wei et al. [24] developed a novel unified control strategy to restrain the DC-link ripple for VSC-HVDC under unbalanced grid conditions. In addition, different power flow algorithms for multi-terminal VSC-HVDC system have been proposed [25–26]. Li et al. [27] investigated a coordinated control strategy of series multi-terminal VSC-HVDC for offshore wind farm.

At present, in most of VSC-HVDC projects, trial and error method and empirical method are widely employed to choose the PI regulator parameters, which require high skills and experience in the system debugging process. Moreover, some further improvement and correction for the regulator are needed in the operation to guarantee the system works on the optimal state. The auto-disturbance rejection control (ADRC) has overcome some disadvantages of PI. And it is researched and successfully used in many fields. Liu investigated the ADRC method on permanent magnet synchronous motor [28]. Piezoelectric multimode vibration control for stiffened plate using ADRC-based was studied in Ref. [29]. Refs. [30–32] applied ADRC to ultrastable optical cavities, a Micro-Electro-Mechanical Systems and unmanned surface vessel course tracking, respectively. Furthermore, in order to suppress nonlinear characteristics of the pseudo-linear system, Ref. [33] raised a control strategy based on ADRC and constructed a least squares support vector machines (LSSVM) inverse model. Shi et al. [34] developed a parameters self-turning of ADRC by support vector machines method applied to improve the maneuverability of air cushion vehicle. Li [35] put forward an improved ADRC controller based on standard support vector machines and applied it to a nonlinear chemical process to adjust pH value. In order to improve ADRC observation accuracy and its response speed, we proposed ADRC improved by least squares support vector machines method, and successfully applied it to permanent magnet synchronous motor both vector control and direct torque control systems [36–37].

As a further study, we in this paper develop our proposed ADRC optimized LSSVM method (improved auto-disturbance rejection control method optimized by least squares support vector machines) to improve the performance of VSC-HVDC system, and particularly our method aims at the rectifier side in the VSC-HVDC system. Specifically, with the input signal of given DC voltage and the output signal of the given d-axis component, we design the ADRC controller for DC voltage outer loop. Then the ADRC optimizing training process is carried out by LSSVM with the input signal of *z*_1_ and the output signal of *z*_2_ in the ADRC controller. The steady state and dynamic performances of the system both can be improved. In addition, we carry out simulations to verify the effectiveness of the proposed method.

From another aspect, the signals in VSC-HVDC system are time series signals. Time series analysis has attracted a great deal of attention from different research fields, and the time series analysis methods have been successfully implemented to solve many challenging problems, such as least-squares method [38–39], fuzzy logic system [40–41], neural networks [42],wavelet analysis [43], Wigner-Ville distribution (WVD) [44–48], adaptive optimal kernel time-frequency representation [49–50], detrended fluctuation analysis [51] and complex network [52–55]. Among them, time-frequency representation, which has been widely applied to analyze non-stationary signals, can simultaneously present the energy characteristics in time and frequency domain. We in this paper employ the time-frequency representation methods (WVD and AOK TFR) to analyze the VSC-HVDC system signals and the results indicate that our proposed method performs better than the traditional method from the perspective of energy distribution in time and frequency plane. The application of time-frequency representation methods to VSC-HVDC system has important implications.

The organization of this paper is as follows: We present the background and current situation about VSC-HVDC technology in the first section. The second section introduces the high frequency transient mathematical model of VSC-HVDC system, which is the basis of the following research. We investigate ADRC theory and design the ADRC controller for DC loop in the third section. In the fourth section, we deeply investigate the ADRC optimized by LSSVM method for constant DC voltage control, and then we get the mathematical model of the control. In the fifth section, we do simulations and use the time-frequency representation methods to analyze the VSC-HVDC system signals to verify the effeteness of the proposed method. We present the conclusions in the last section.

## The High Frequency Transient Mathematical Model of VSC-HVDC System

The high frequency transient mathematical model of VSC-HVDC system is based on the switching function description [56].

Define a binary logic switching function *S*_*k*_:

Sk={12−12  k=a,b,c (1)

where, Sk=−12 means the lower bridge arm in the K bridge arm is conducted, the corresponding upper bridge arm is off. Sk=12 means the upper bridge arm in the K bridge arm is conducted, the corresponding lower bridge arm is off.

The VSC voltage equation of AC three-phase is established applying KVL law.

$$\left\{ \begin{array}{l} u_{ca}=L\frac{di_{a}}{dt}+Ri_{a}+u_{sa}\\ u_{cb}=L\frac{di_{b}}{dt}+Ri_{b}+u_{sb}\\ u_{cc}=L\frac{di_{c}}{dt}+Ri_{c}+u_{sc} \end{array}\right.$$ (2)

where, *u*_*ca*_,*u*_*cb*_,*u*_*cc*_ are the three phase voltage instantaneous values of the AC power system, respectively.*u*_*sa*_,*u*_*sb*_,*u*_*sc*_ are the three phase voltage instantaneous values of the convertor, respectively. *i*_*a*_,*i*_*b*_,*i*_*c*_ are the three phase currents, respectively. *L* is the converter transformer inductance. *R* is the equivalent resistance.

Define:

$$u_{ck}=S_{k}U_{dc}$$ (3)

where, *U*_*dc*_ is the DC voltage.

Combining Eq (2) and Eq (3), we can obtain the following equation.

$$\left[\begin{array}{c} u_{ca}\\ u_{cb}\\ u_{cc} \end{array}\right]=\left[\begin{array}{c} S_{a}U_{dc}\\ S_{b}U_{dc}\\ S_{c}U_{dc} \end{array}\right]=L\frac{d}{dt}\left[\begin{array}{c} i_{a}\\ i_{b}\\ i_{c} \end{array}\right]+R\left[\begin{array}{c} i_{a}\\ i_{b}\\ i_{c} \end{array}\right]+\left[\begin{array}{c} u_{sa}\\ u_{sb}\\ u_{sc} \end{array}\right]$$ (4)

Then the following form can be obtained.

$$\frac{d}{dt}\left[\begin{array}{c} i_{a}\\ i_{b}\\ i_{c} \end{array}\right]=\frac{U_{dc}}{L}\left[\begin{array}{c} S_{a}\\ S_{b}\\ S_{c} \end{array}\right]-\frac{R}{L}\left[\begin{array}{c} i_{a}\\ i_{b}\\ i_{c} \end{array}\right]-\frac{1}{L}\left[\begin{array}{c} u_{sa}\\ u_{sb}\\ u_{sc} \end{array}\right]$$ (5)

At any moment, there are three conducted switch tube in VSC. If Sk=12, then Sk+12=1. It means that the upper bridge arm in the K bridge arm is conducted. Therefore the current of the upper bridge arm is ik(Sk+12)=ik. If Sk=−12, then Sk+12=0. It means that the lower bridge arm in the K bridge arm is conducted. Consequently, the current of the upper bridge arm is 0, and the DC current *I*_*dc*_ can be described by the following equation:

$$I_{dc}=i_{a}(S_{a}+\frac{1}{2})+i_{b}(S_{b}+\frac{1}{2})+i_{c}(S_{c}+\frac{1}{2})$$ (6)

The following Eq (7) is derived through applying Kirchhoff's current law (KCL) on the capacitance positive node side.

$$C\frac{dU_{dc}}{dt}=I_{dc}-I_{dl}$$ (7)

where, *I*_*dl*_ is the load current.

The following equation can be derived by substituting Eq (6) into Eq (7).

$$\frac{dU_{dc}}{dt}=\frac{1}{C}\left(i_{a}(S_{a}+\frac{1}{2})+i_{b}(S_{b}+\frac{1}{2})+i_{c}(S_{c}+\frac{1}{2})\right)-\frac{I_{dl}}{C}$$ (8)

Eq (9) presents the equation of state shown in follows, in which *X* = [*i*_*a*_,*i*_*b*_,*i*_*c*_,*U*_*dc*_]^*T*^.

$$\dot{X}=AX+BE$$ (9)

where,

$$A=\left[\begin{array}{cccc} -\frac{R}{L} & 0 & 0 & \frac{S_{\text{a}}}{L}\\ 0 & -\frac{R}{L} & 0 & \frac{S_{b}}{L}\\ 0 & 0 & -\frac{R}{L} & \frac{S_{c}}{L}\\ \frac{\left(S_{a}+\frac{1}{2}\right)}{C} & \frac{\left(S_{b}+\frac{1}{2}\right)}{C} & \frac{\left(S_{c}+\frac{1}{2}\right)}{C} & 0 \end{array}\right];B=\left[\begin{array}{cccc} -\frac{1}{L} & 0 & 0 & 0\\ 0 & -\frac{1}{L} & 0 & 0\\ 0 & 0 & -\frac{1}{L} & 0\\ 0 & 0 & 0 & \frac{I_{dl}}{C} \end{array}\right];E=\left[\begin{array}{l} u_{sa}\\ u_{sb}\\ u_{sc}\\ 1 \end{array}\right].$$

## The ADRC Controller for DC Loop Design

The PI control method which has been widely used has the advantages such as simple construction, but it also has some shortages, such as:

1. PI control is a kind of linear combination, leading to the fact that it is difficult to meet high performance requirement for a nonlinear system in the practical engineering.
2. When the system is subject to random disturbances, PI control method usually cannot achieve expected effect.

So in order to carry forward the benefits of PI control, and overcome its disadvantages, Han Jingqing has developed a new control method, named auto-disturbance rejection control [57]. This control method allows estimating system disturbance real-timely and timely feedforward compensate it. Meanwhile, he proposed a reasonable nonlinear function to improve the PI linear combination disadvantage. The following is the ADRC working principle and ADRC controller design for DC loop.

**Fig 1:**
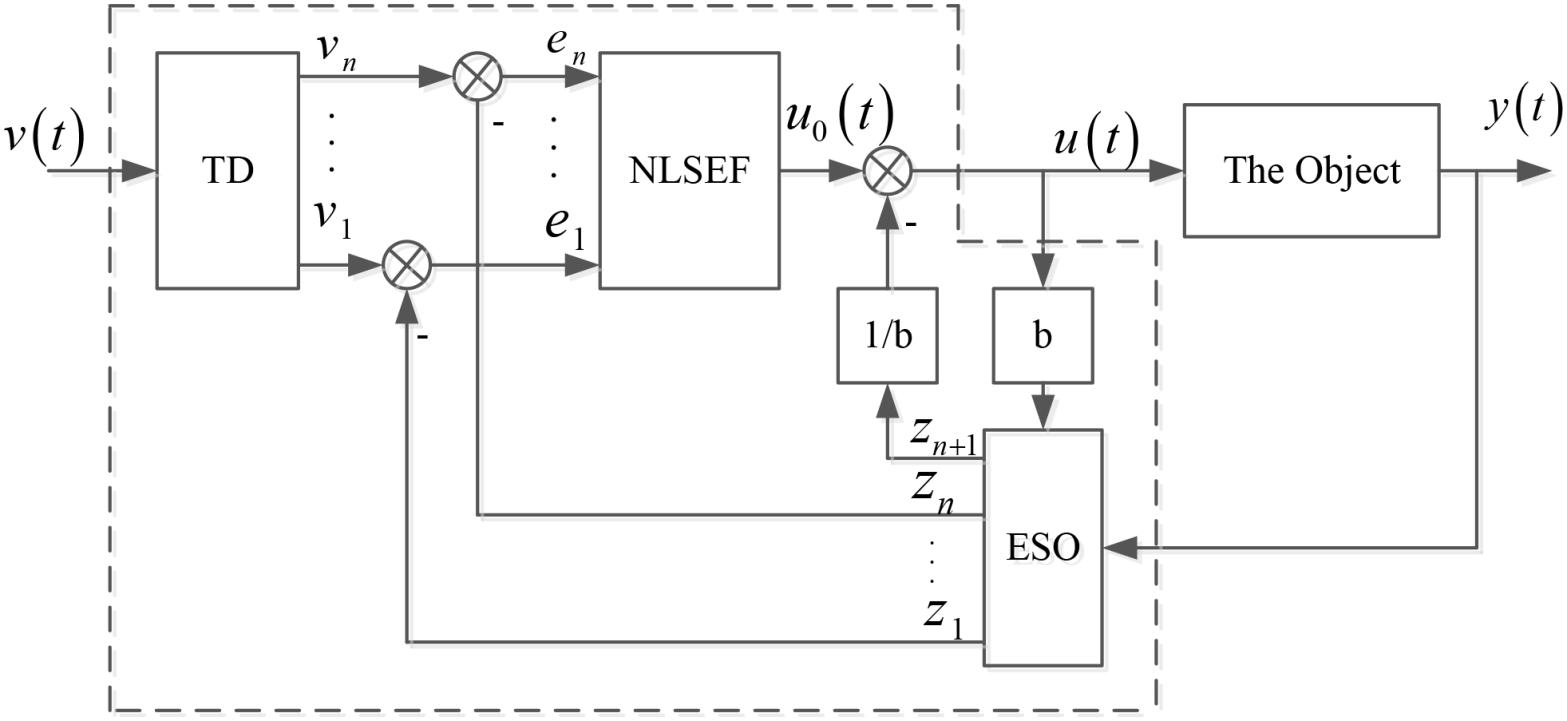
The diagram of ADRC controller.

ADRC is composed of the track—differentiator (TD), extended state observer (ESO) and nonlinear state error feedback control rate (NLSEF) [58–60]. The diagram of ADRC controller is shown in Fig 1, where, *v*(*t*) is the input signal; *v*_1_ is the transient process of *v*(*t*); *v*_*n*_ is the n-1 order differential signal; *y*(*t*) is the output signal of the control object; *z*_1_….*z*_*n*_ are the state variables estimated by ESO, respectively; *z*_n+1_ is the system disturbance estimated by the ESO; *e*_1_….*e*_*n*_ are the errors of *v*_1_…..*v*_*n*_ and *z*_1_…..*z*_*n*_, respectively; *u*_0_(*t*) is the initial control signal from NLSEF; *u*(*t*) is the final control signal; *b* is the compensating factor.

We here take a first-order system as an example to illustrate its principle. The state equation of a first-order system is as follows:

$$\left\{ \begin{array}{l} \overset{\cdot }{x}=f\left(x,t\right)+bu\\ y=x \end{array}\right.$$ (10)

where, *f*(*x*,*t*) is an unknown function and *u* is the system control signal.

Then the track—differentiator model in ADRC can be described as follows:

$$\left\{ \begin{array}{l} e_{0}=v_{1}-v\\ {\overset{\cdot }{v}}_{1}=-fst\left(e_{0},r,T\right) \end{array}\right.$$ (11)

where *fst*(*e*_0_,*r*,*T*) is $\left\{ \begin{array}{l} d=rT;\text{​ }d_{0}=dT;\\ y_{TD}=e_{o};\;a_{0}={\left(d^{2}+8r\left|y_{TD}\right|\right)}^{1/2};\\ a=\left\{ \begin{array}{l} \left(a_{0}-d\right)/2\;\left|y_{TD}\right|>d_{0}\\ y_{TD}/T\;\left|y_{TD}\right|\leq d_{0}\; \end{array}\right.\\ fst=-\left\{ \begin{array}{l} ra/d\;\left|a\right|\leq d\\ r\text{sgn}\left(a\right)\;\left|a\right|>d \end{array}\right. \end{array}\right.$

*v* is the input signal of ADRC; *v*_1_ is the tracking signal of *v*; *r* is tracking speed factor; *T* is sample time.

The core of ADRC is the extended state observer (ESO). The output of the controlled object *y*(*t*) is tracked, and the derivative of state variables and disturbance are estimated. The disturbance is compensated previously fed.

Then function *f*(*x*,*t*) is taken as disturbance signals. Take ${\overset{\cdot }{x}}_{2}=g\left(x,t\right)$, thus the following can be got.

$$\left\{ \begin{array}{l} {\overset{\cdot }{x}}_{1}=f\left(x,t\right)+bu\\ {\overset{\cdot }{x}}_{2}=g\left(x,t\right)\\ y=x_{1} \end{array}\right.$$ (12)

Then mathematical model of extended state observer is as follows.

$$\left\{ \begin{array}{l} e=z_{1}-y\\ {\overset{\cdot }{z}}_{1}=z_{2}-\beta_{01}fal\left(e,\alpha_{1},\delta \right)+bu\left(t\right)\\ {\overset{\cdot }{z}}_{2}=-\beta_{02}fal\left(e,\alpha_{2},\delta \right) \end{array}\right.$$ (13)

where *y* is the output signal of the object; *z*_1_ is the tracking signal of *y*; *z*_2_ is the estimation value of disturbance; *α*_1_,*α*_2_ is nonlinear factor; *δ* is filter factor; *β*_01_,*β*_02_ is correction gain of output error; *fal*(*e*,*α*,*δ*) is nonlinear function, and its expression is as follows: $fal\left(e,\alpha ,\delta \right)=\{\begin{array}{l} \frac{e}{\delta^{\alpha -1}},\;\left|e\right|\leq \delta \\ {\left|e\right|}^{\alpha }sign\left(e\right),\;\left|e\right|>\delta \; \end{array}.$

The mathematical model of nonlinear state error feedback control in ADRC for system can be described as follows:

$$\left\{ \begin{array}{l} e_{1}=v_{1}-z_{1}\\ u_{0}=\beta_{1}fal\left(e_{1},\alpha_{3},\delta_{1}\right)\\ u=u_{0}-z_{2}/b \end{array}\right.$$ (14)

where *β*_1_ is the gain; *u*_0_ is initial control signal from NLSEF; *u* is the control signal of ADRC.

**Fig 2:**
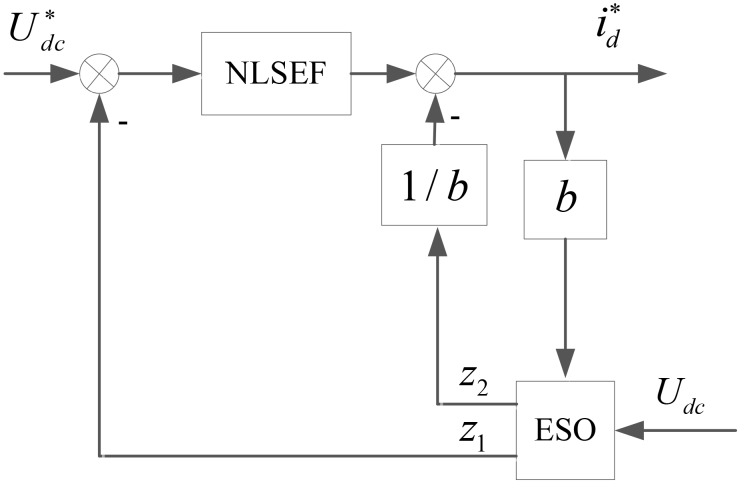
The ADRC controller diagram for the constant DC voltage control.

In order to improve the system response speed, the TD part in ADRC controller is ignored in this paper. In the rectifier side for VSC-HVDC system, according to the mathematic model shown in Eqs (13) and (14), the ADRC controller for DC voltage loop is designed with the input signal of $U_{DC}^{*}$ and the output signal of the given d-axis component $i_{d}^{*}$. The ADRC controller diagram is shown in Fig 2. Its working principle is as follows: In accordance with the above ADRC theory, the output signals of ESO are *z*_1_ and *z*_2_, where *z*_1_ is the tracking signal of the actual DC voltage value *U*_DC_, and *z*_2_ is the estimated value of the system disturbance. Then we can get the error between the given DC voltage value $U_{DC}^{*}$ and its actual value *U*_DC_. With this error as the input signal of NLSEF, and substitute it into the mathematical model of NLSEF shown in Eq (14), we can get the initial control signal from NLSEF which is *u*_0_. After that, with the feedforward compensation of the estimated disturbance *z*_2_, the control signal of ADRC $i_{d}^{*}$ can be obtained.

## The ADRC Optimized by LSSVM for Constant DC Voltage Control

The least squares support vector machines (LSSVM), which is developed on the basis of the statistical theory, is an extension of support vector machine (SVM). It transforms the quadratic programming problem in the training process of the standard SVM into solving linear equations problem by least squares method. In the algorithm, the optimization index uses square term. Under the above improvement, the computational complexity is dramatically reduced, and the efficiency of training can be greatly improved. In addition, LSSVM has remained the strong generalization ability and the global optimal ability of the standard SVM. This method has well solved some problems such as the small sample, nonlinearity, high dimension and local minimum point, and has been widely used in pattern recognition, signal processing and time series analysis fields. On account of different problems, LSSVM algorithm is usually divided into two categories: one is the classification problem; and another kind is the regression problems. In this paper, we use LSSVM to solve the nonlinear regression problem.

Define: $T=\left\{(x_{k},y_{k})|k=1,2,3,\cdots ,n\right\},x_{k}\in R^{n},y_{k}\in R$ is the training sample data. *x*_*k*_ is the input data. *y*_*k*_ is the output data. The optimization problem in the raw space (*w* space) can be described as follows [61–63].

$$\text{min}C\sum_{i=1}^{n}(\xi_{i}+\xi_{i}^{*})+\frac{1}{2}w_{i}^{2}$$ (15)

Subject to

$$\left\{ \begin{array}{l} f(x)=(w,x)+b\\ y_{i}-f(x_{i})-e\leq \xi_{i}\\ f(x_{i})-y_{i}-e\leq \xi_{i}^{*}\\ \xi_{i}^{*}\geq 0 \end{array}\right.$$ (16)

Use the error sum of squares instead of slack variable. And let inequality constraints be equality constraints. The LSSVM regression optimization is shown in Eq (17).

$$\underset{w,b,e}{\text{min}}J(w,e)=\frac{1}{2}w^{T}w+\frac{1}{2}\gamma \sum_{k=1}^{N}e_{k}^{2}$$ (17)

The constraint condition is as follows.

$$y_{k}=w^{T}w\varphi (x_{k})+c+e_{k},\;k=1,2,\cdots N$$ (18)

where, *φ*():*R* ^*n*^→*R* ^*m*^ is the kernel space mapping function; *w*ϵ*R* ^*m*^ is the weight vector; *e*_*k*_ **ϵ** *R* is the error vector; *c* is the offset.

The Lagrange function is constructed in Eq (19).

$$L(w,b,e;\alpha )=J(w,e)-\sum_{k=1}^{N}\alpha_{k}\left\{w^{T}\varphi (x_{k})+b+e_{k}-y_{k}\right\}$$ (19)

where, *α*_*k*_ϵ*R* is the Lagrange factor.

Eq (20) is derived by derivation of Eq (19).

$$\left\{ \begin{array}{l} \frac{\partial L}{\partial w}=0,\;w=\sum_{k=1}^{N}\alpha_{k}\varphi (x_{k});\\ \frac{\partial L}{\partial b}=0,\;\sum_{k=1}^{N}\alpha_{k}=0;\\ \frac{\partial L}{\partial e_{k}}=0,\;\alpha_{k}=\gamma e_{k};\\ \frac{\partial L}{\partial \alpha_{k}}=0,\;w^{T}\varphi (x_{k})+b+e_{k}-y_{k}=0 \end{array}\right.$$ (20)

The matrix equation is obtained.

$$\left(\begin{array}{cc} 0 & 1_{v}^{T}\\ 1_{v} & \Omega +\frac{1}{\gamma }l \end{array}\right)\left(\begin{array}{c} b\\ \alpha \end{array}\right)=\left(\begin{array}{c} 0\\ y \end{array}\right)$$ (21)

where,*y* = (*y*_1_,*y*_2_,…,*y*_n_); 1_*v*_ = (1,1,…,1); *α* = (*α*_1_,*α*_2_,…,*α*_n_); Ω = {Ω_*kl*_}_*N×N*_ Ω_*kl*_ = *φ*(*x*_*k*_)^T^ *φ*(*x*_*l*_), *k*,*l* = 1,2,3…,*N*.

According to mercer condition, the mapping function *φ* and the kernel function *k*(·,·) exist, which satisfy the following equation.

$$K(x_{k},x_{l})=\varphi {\left(x_{k}\right)}^{T}\varphi (x_{l})$$ (22)

Therefore, the optimization estimation of LSSVM is shown in Eq (23).

$$y(x)=\sum_{k=1}^{N}\alpha_{k}K(x,x_{k})+b$$ (23)

LSSVM algorithm adopts equality constraint in the process of regression. The solving optimization eventually converts into solving linear equation, and the calculation process is greatly simplified.

In the ADRC controller for the constant DC voltage control, the training process is carried out with the input signal of *z*_1_ and the output signal of *z*_2_. From the above, the optimal regression LSSVM model is obtained. After that, the optimal model is used to replace the ADRC controller. The trained LSSVM model can estimate part of the system disturbance *g*_*SVM*_ according to the input signal *z*_1_ real-timely. The rest disturbance is estimated by the ESO. Then we can obtain the sum of the system disturbance, which is feedforward compensated after subsequent operations in real-time. The ADRC optimized by LSSVM controller can improve system response speed and the observation accuracy of ADRC. The system anti-interference ability is further improved. The mathematical model of the ADRC optimized by LSSVM for the constant DC voltage control can be described by Eqs (24) and (25).

ESO:

$$\left\{ \begin{array}{l} e=z_{1}-U_{dc}\\ {\dot{z}}_{1}=z_{2}-\beta_{01}fal(e,\alpha_{1},\delta_{1})+bi_{d}^{*}+g_{SVM}\\ \overset{\cdot }{z_{2}}=-\beta_{02}fal(e,\alpha_{1}/2,\delta_{1}) \end{array}\right.$$ (24)

NLSEF:

$$\left\{ \begin{array}{l} e_{1}=U_{dc}^{*}-z_{1}\\ i_{d}^{*}=\beta_{1}fal\left(e_{1},\alpha_{2},\delta_{2}\right)-\left(z_{2}+g_{SVM}\right)/b \end{array}\right.$$ (25)

## Simulations and Time-Frequency Representation Analysis

**Fig 3:**
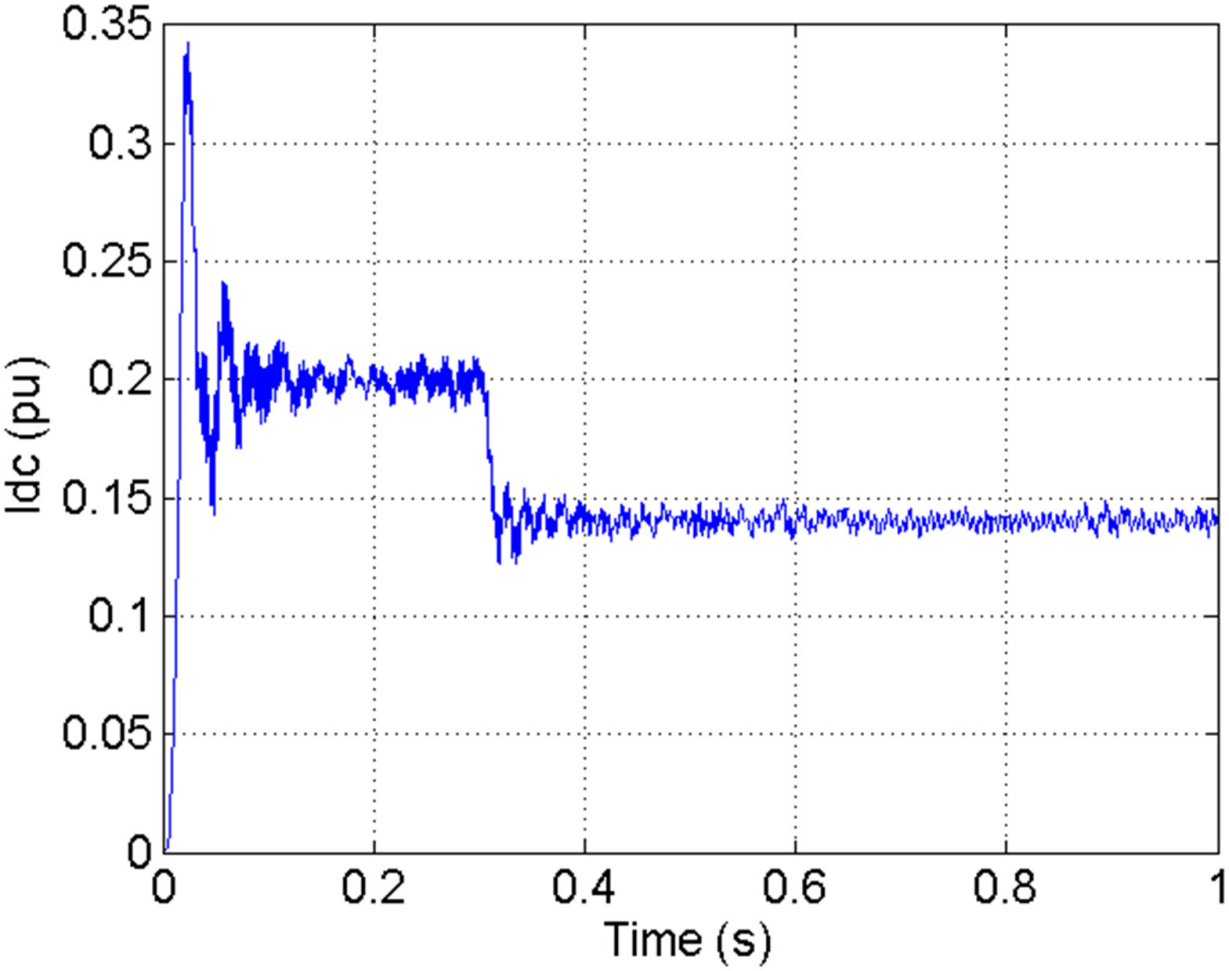
The DC current wave for PI method.

**Fig 4:**
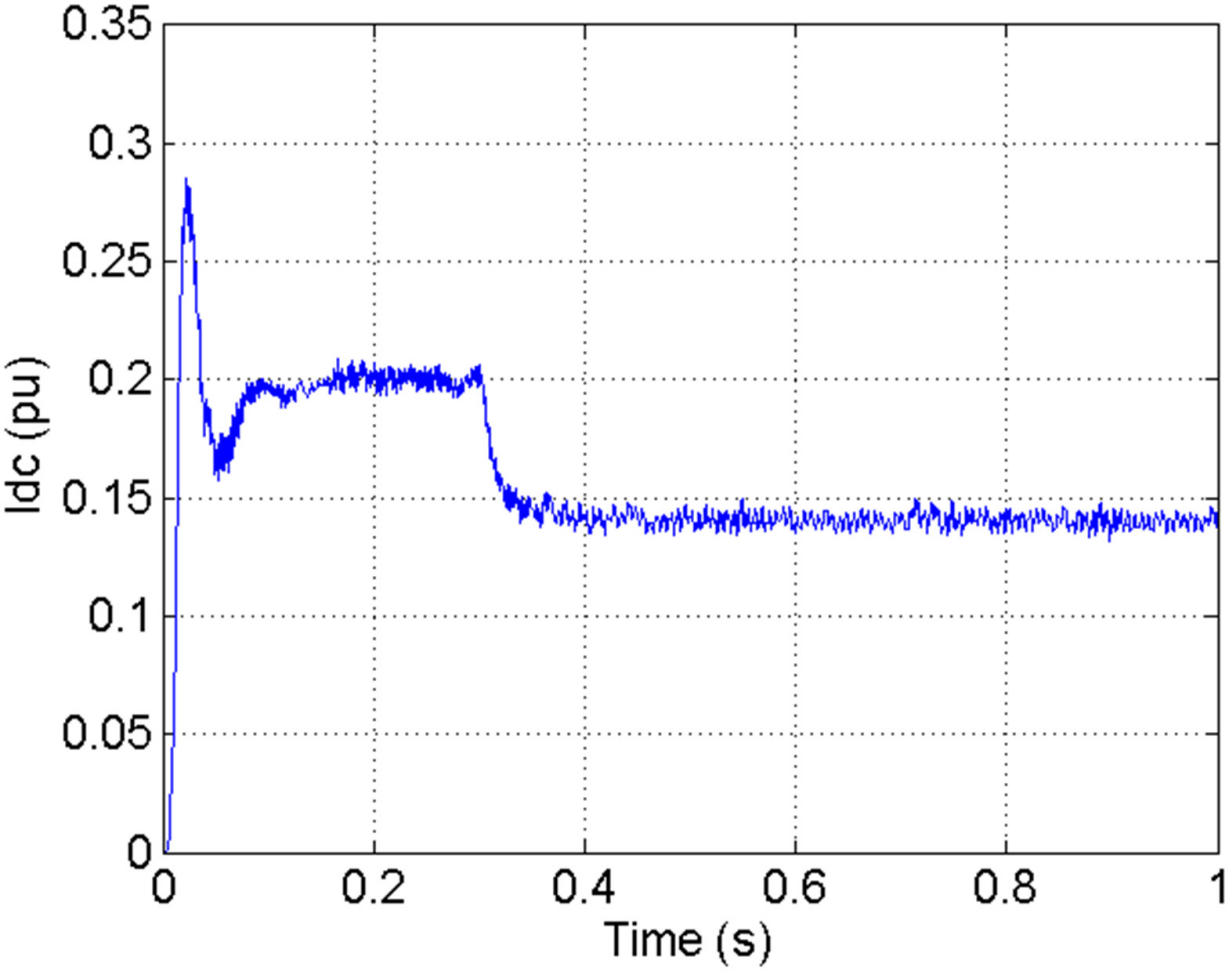
The DC current wave for our proposed method.

**Fig 5:**
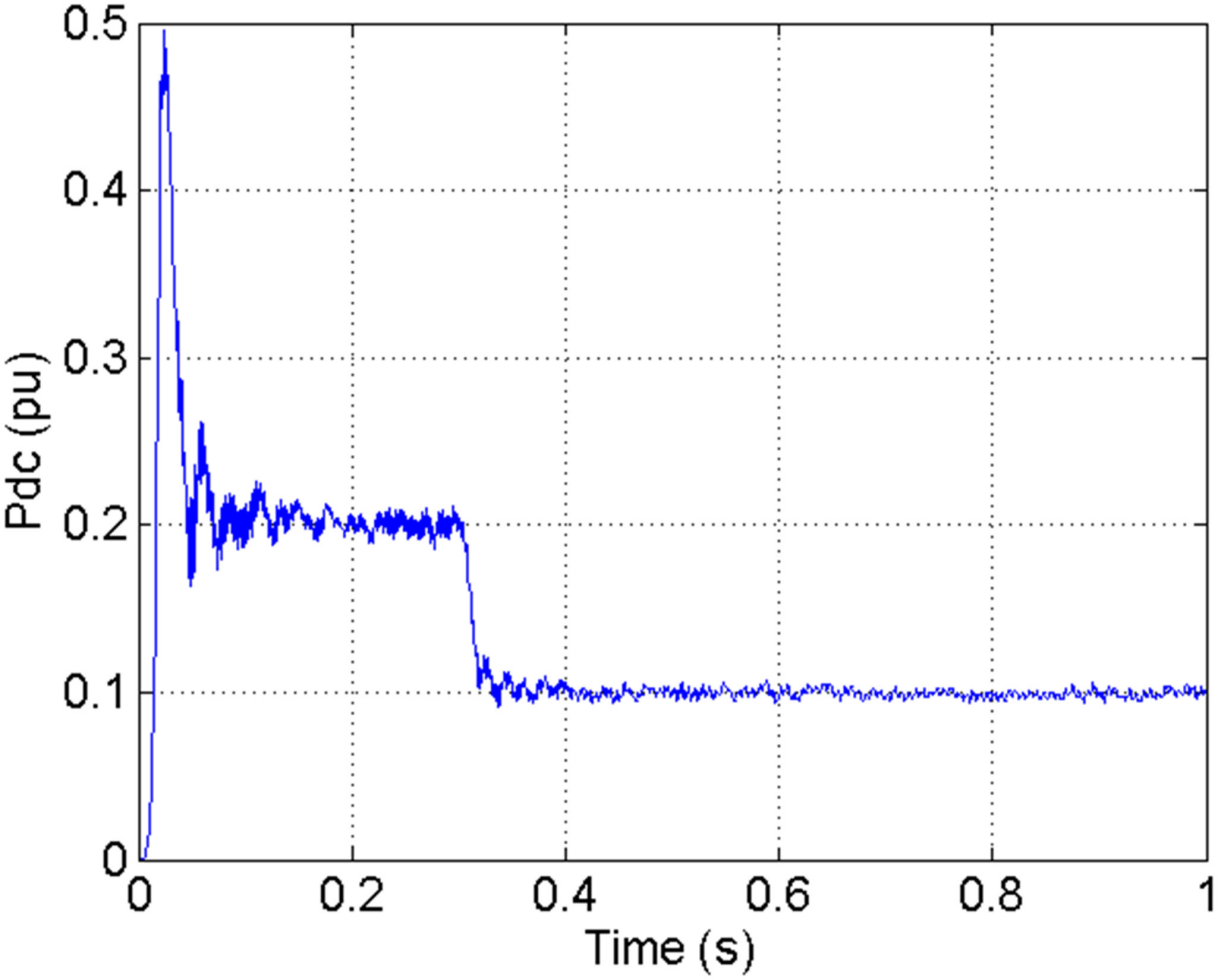
The transmitted power wave in DC side for PI method.

**Fig 6:**
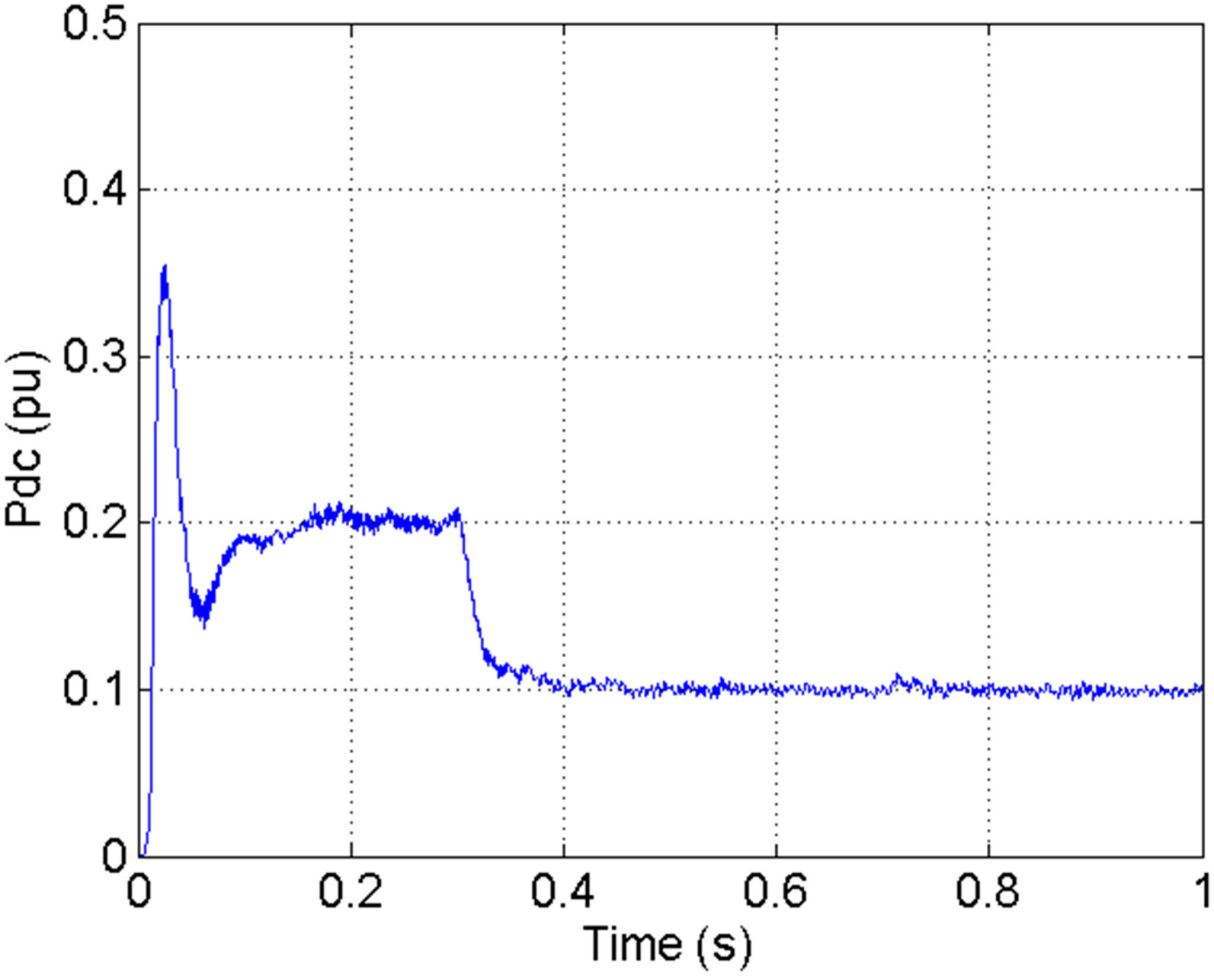
The transmitted power wave in DC side for our proposed method.

**Fig 7:**
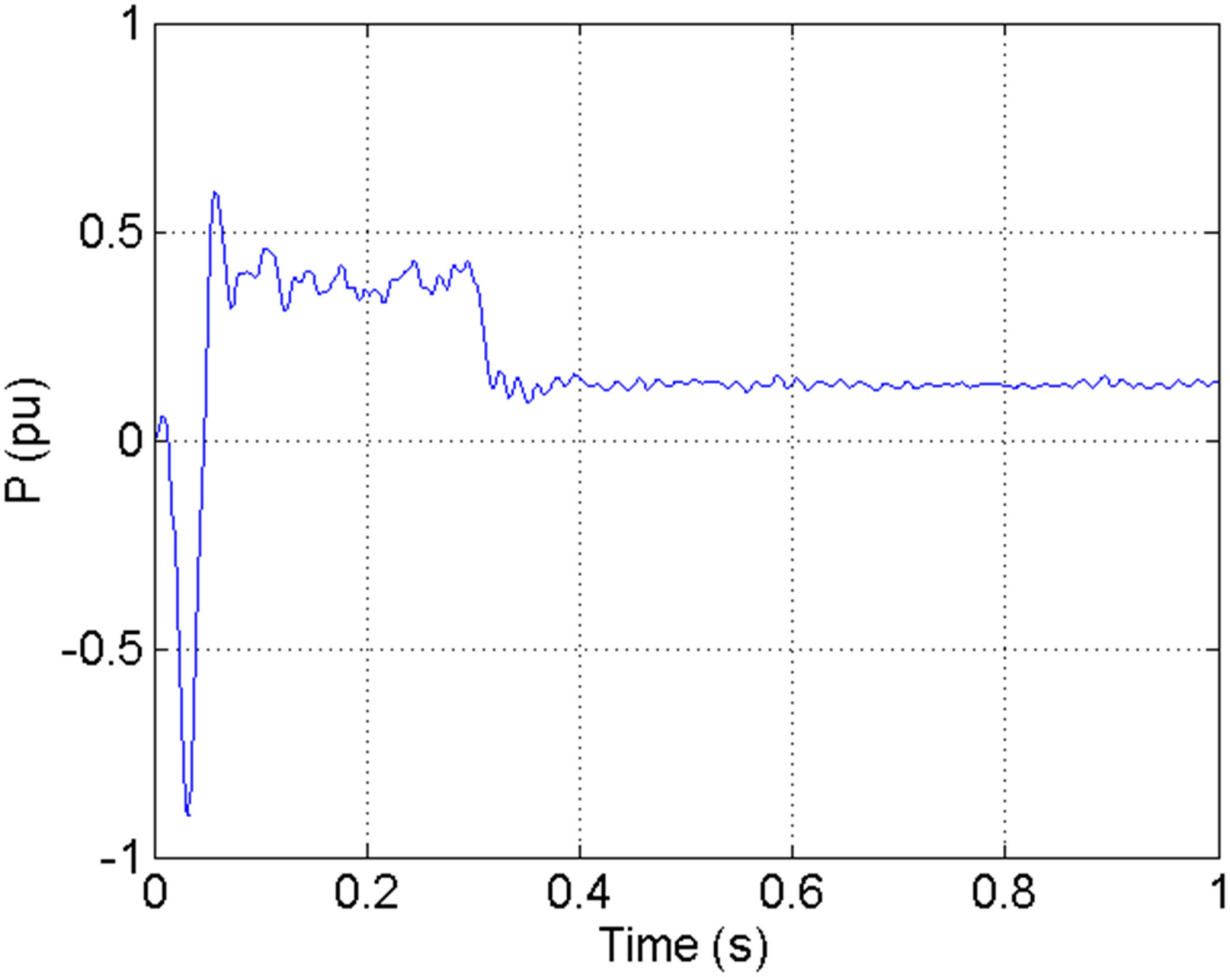
The active power wave for PI method.

**Fig 8:**
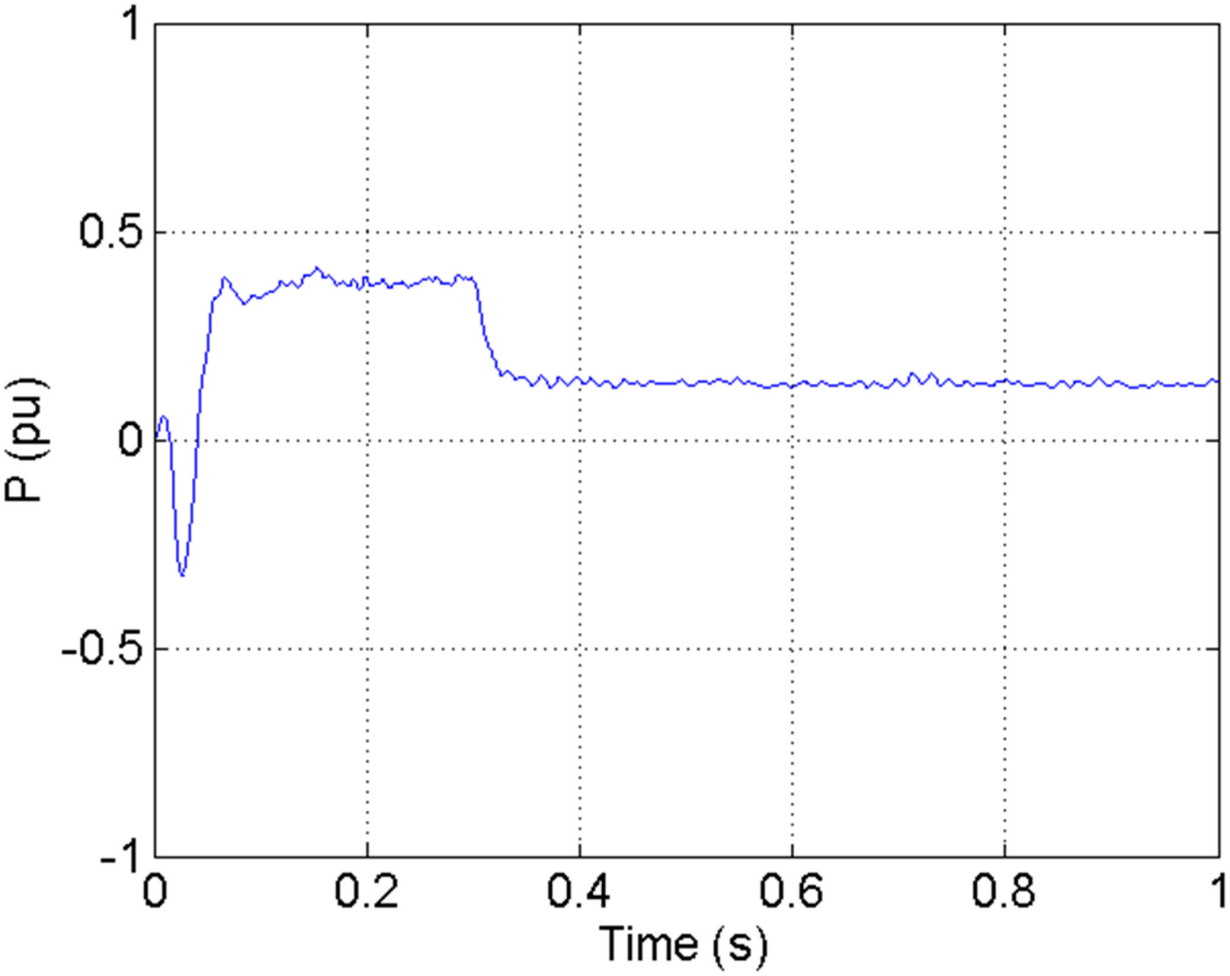
The active power wave for our proposed method.

**Fig 9:**
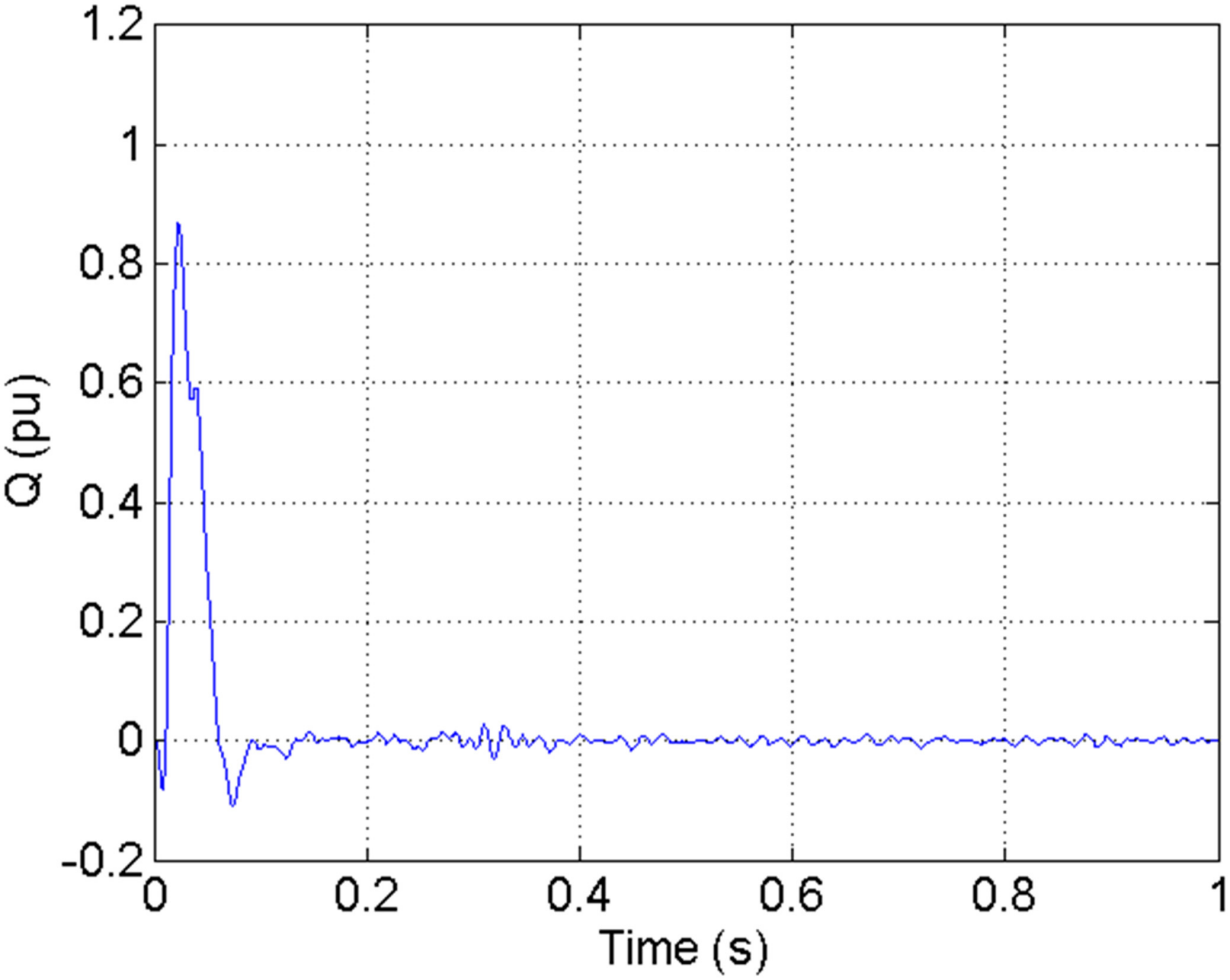
The reactive power wave for PI method.

**Fig 10:**
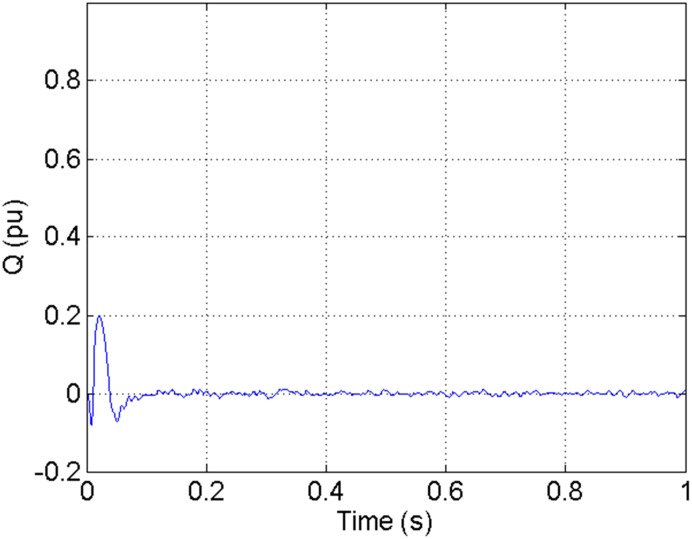
The reactive power wave for our proposed method.

According to the theory above, we using MATLAB/SIMULINK construct the VSC-HVDC system simulation model. In particular, we carry out a contrastive simulation for the proposed method and traditional PI method, with the purpose of demonstrating the advantage of the proposed method in VSC-HVDC system. In the simulation model, the VSC-HVDC system simulation parameters are shown in Table 1. In the whole simulation process, per-unit data is adopted. The reference values are selected as follows: The reference value for the AC power voltage is 100 kV; the capacity reference value is 200MVA; the reference value for DC voltage is 200 kV. The simulation condition is as follows: the given DC voltage is 1pu (per unit), and it changes to 0.7pu at 0.3s. The given reactive power is 0. The rectifier simulation results for our proposed method and PI method are shown in Figs 3 and 4, Figs 5 and 6, Figs 7 and 8, Figs 9 and 10.

**Table 1:** The system simulation parameters.

parameters	values
The equivalent inductance	55.6(mH)
The equivalent resistance	0.3(Ω)
The DC bus capacitors	3000(μF)
The given DC voltage	200(kV)
The AC power voltage	100(kV)
The grid frequency	50(Hz)
Resistance in DC	0.6(Ω)

From the above simulation we can obtain the VSC-HVDC system signals. We use time-frequency representation methods to analyze the signals to indicate advantages of our proposed method from another perspective of energy distribution in time-frequency plane. We use Wigner-Ville distribution (WVD) and adaptive optimal kernel time-frequency representation (AOK TFR) to analyze the signals [64]. The WVD of a signal *s*(*t*) is

$$W_{x}(t,f)=\int_{-\infty }^{+\infty }X(f+\frac{v}{2})X*(f-\frac{v}{2})e^{-\text{j}2\pi tv}\text{d}v\;$$ (26)

where *X*(∙) is the Fourier transform of signal *x*(∙), * denotes conjugation, and signal *x*(*t*) is the Hilbert transform of real signal *s*(*t*), for discrete-time signal *s*(*n*), the discrete Wigner-Ville distribution is

$$W(n,k)=2\sum_{m=-(N-1)/2}^{(N-1)/2}x(n+m)x^{*}(n-m)e^{-\text{j}4\pi mk/N}$$ (27)

where *n*, *k* and *m* is the discrete variable corresponding to continues variable of *t*, *f* and *τ*, respectively.

The adaptive optimal kernel time-frequency representation can be expressed as

$$P(t,f)=\iint A(t,\tau ,\upsilon )\Phi (\tau ,\upsilon )e^{-j2\pi (t\upsilon +\tau f)}d\tau d\upsilon$$ (28)

where Φ(*τ*,*υ*) is a kernel function for generating AOK TFR,

$$\Phi (\tau ,\upsilon )=e^{-r^{2}/2\sigma^{2}(\psi )}$$ (29)

*σ*(*Ψ*) controls the extension of Gaussian kernel at *Ψ* direction, and *r* ^2^ = *τ* ^2^+*υ* ^2^, where *τ* and *υ* are the time-delay and frequency shift, respectively. *A*(*t*,*τ*,*υ*) is a window signal which can be defined as

$$A(t,\tau ,\upsilon )=\int s^{*}(u-\frac{\tau }{2})\cdot \omega^{*}(u-t-\frac{\tau }{2})\cdot s(u+\frac{\tau }{2})\cdot \omega (u-t-\frac{\tau }{2})\cdot e^{j\upsilon u}du$$ (30)

**Fig 11:**
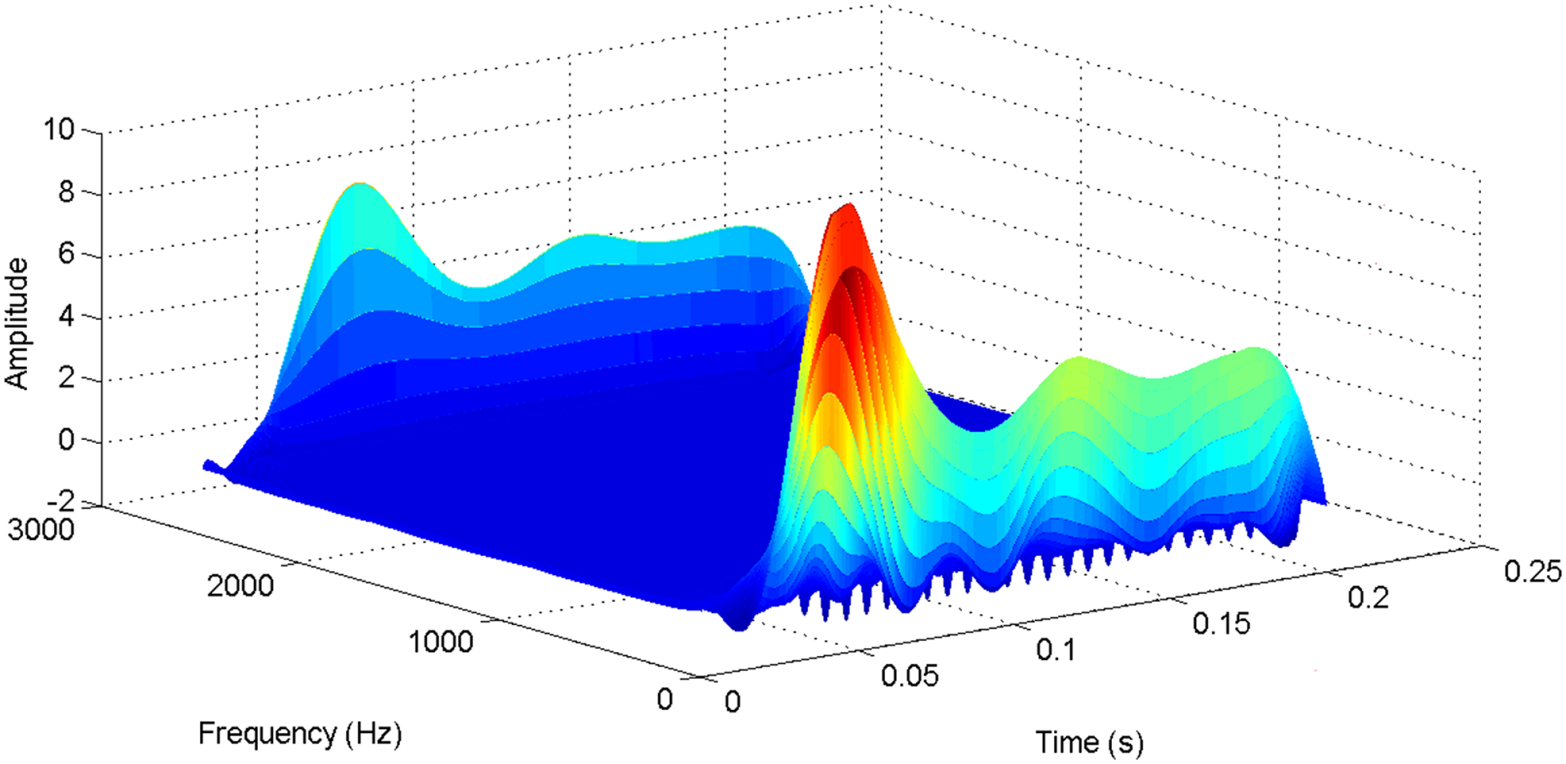
The WVD result of DC current signal for PI method.

**Fig 12:**
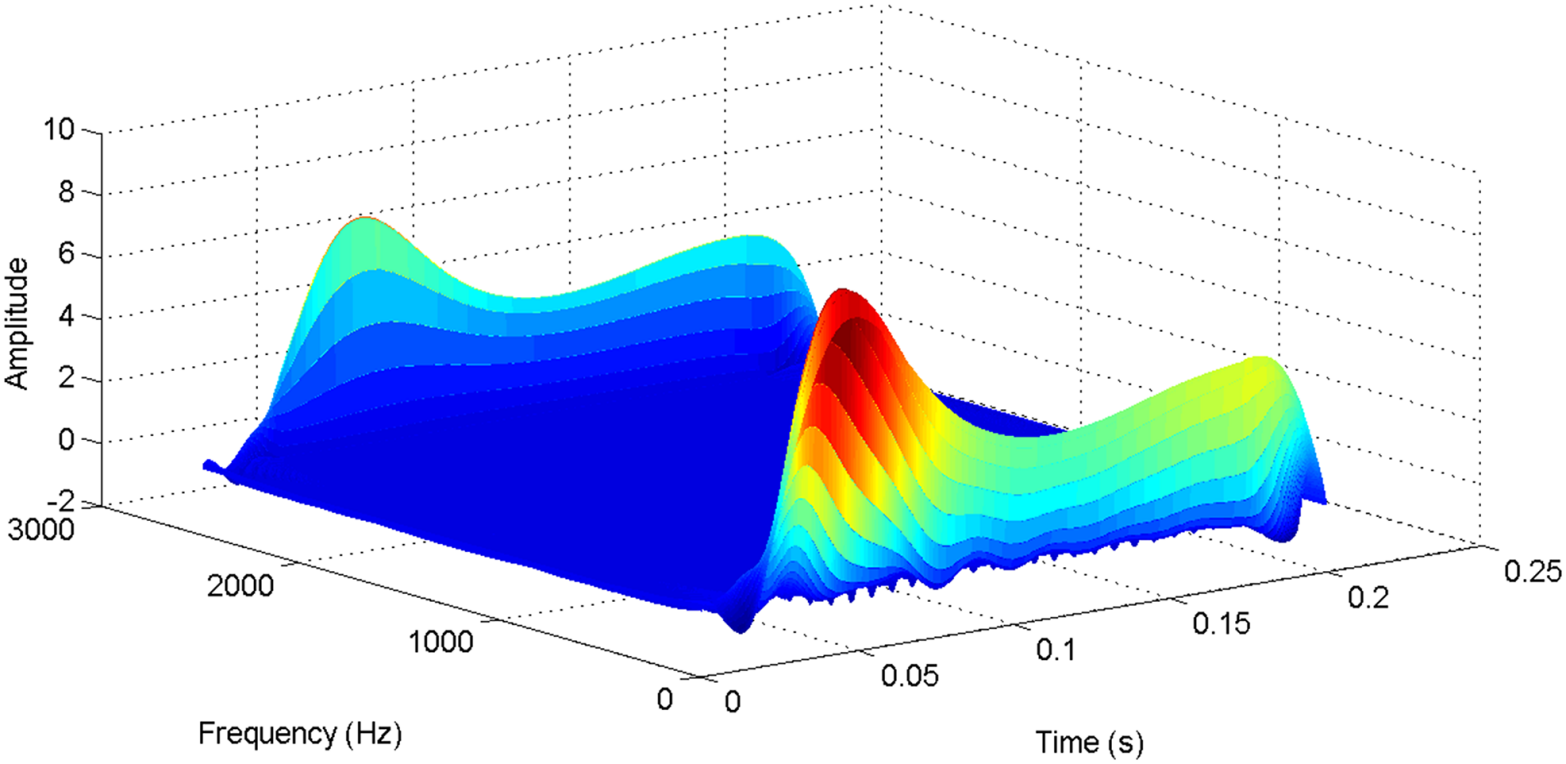
The WVD result of DC current signal for our proposed method.

**Fig 13:**
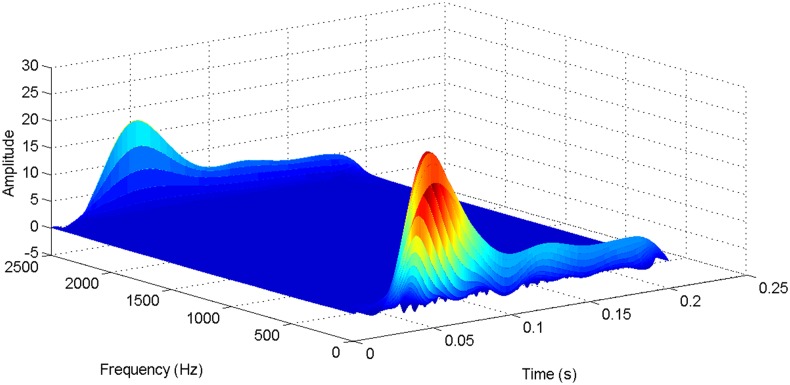
The WVD result of transmitted power signal in DC side for PI method.

**Fig 14:**
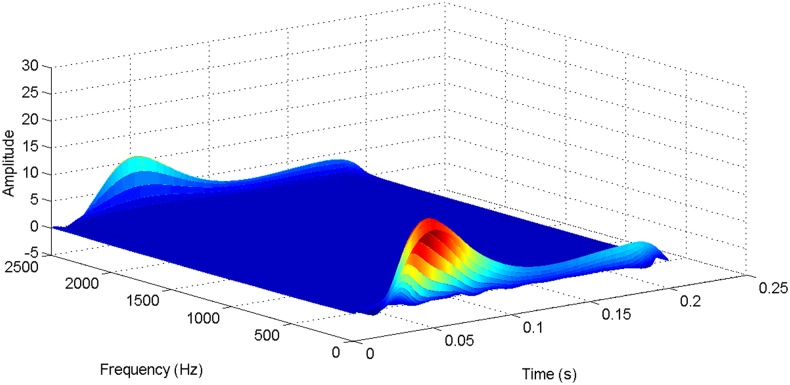
The WVD result of transmitted power signal in DC side for our proposed method.

**Fig 15:**
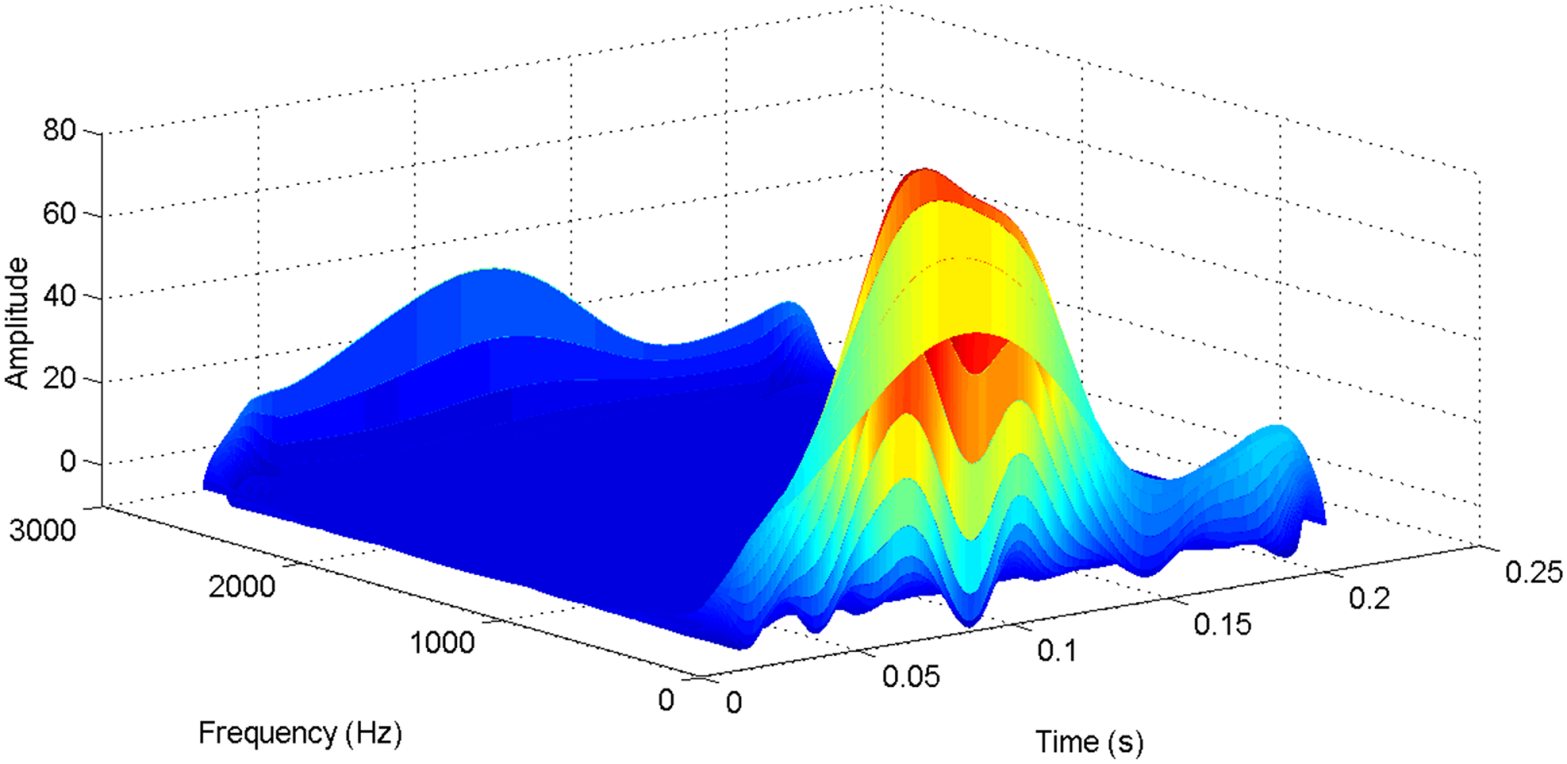
The WVD result of active power signal for PI method.

**Fig 16:**
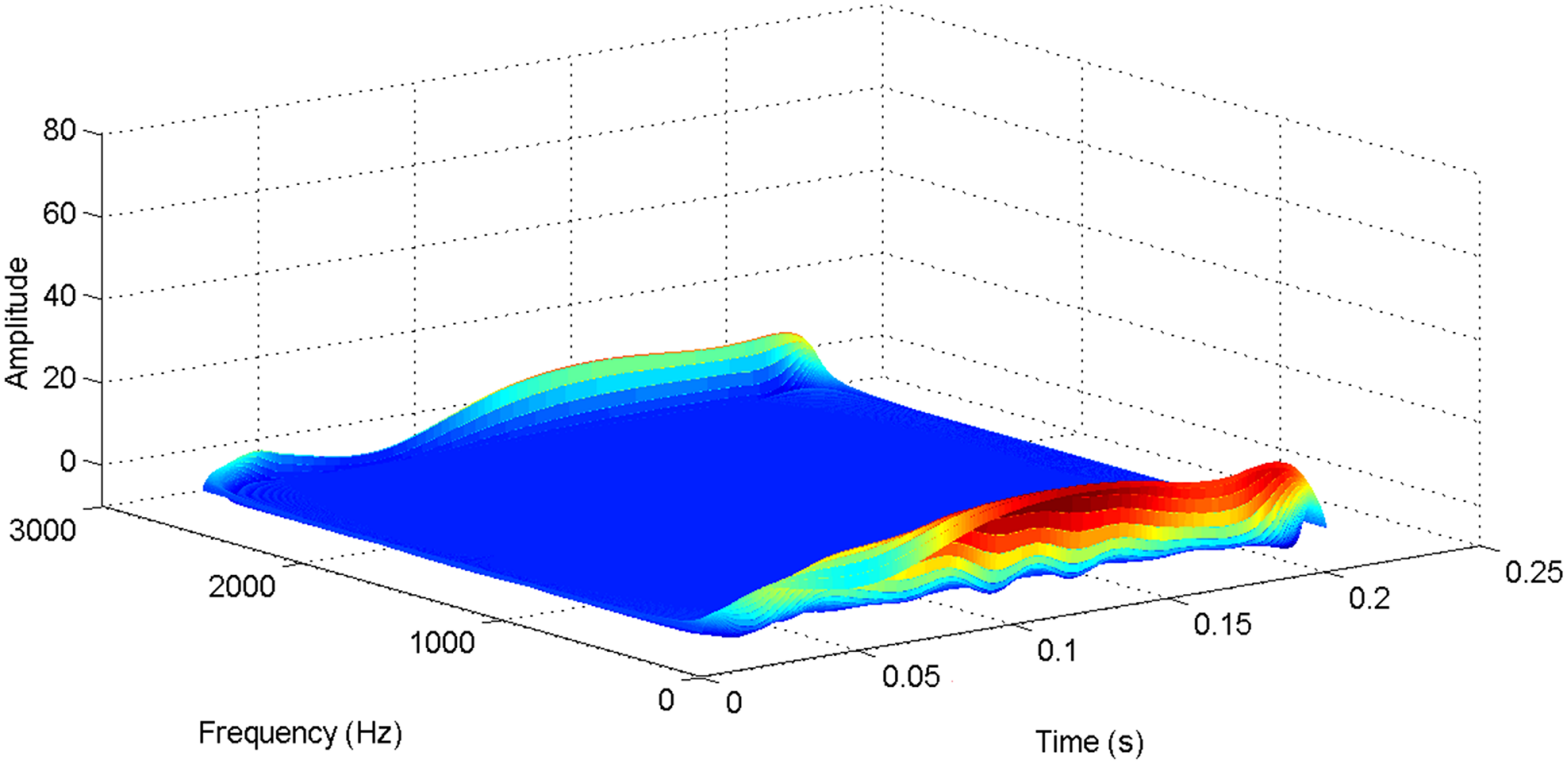
The WVD result of active power signal for our proposed method.

**Fig 17:**
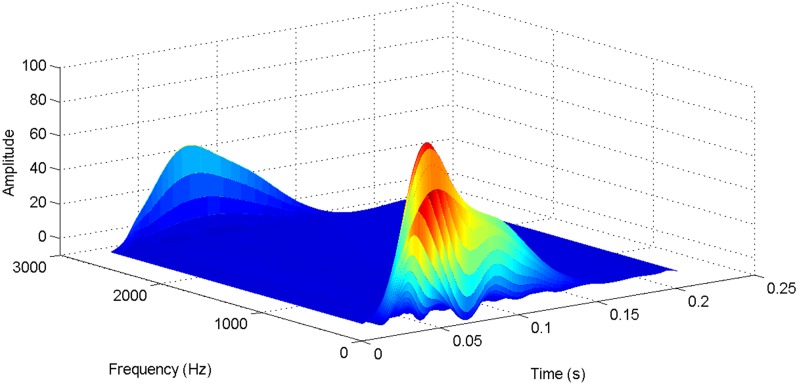
The WVD result of reactive power signal for PI method.

**Fig 18:**
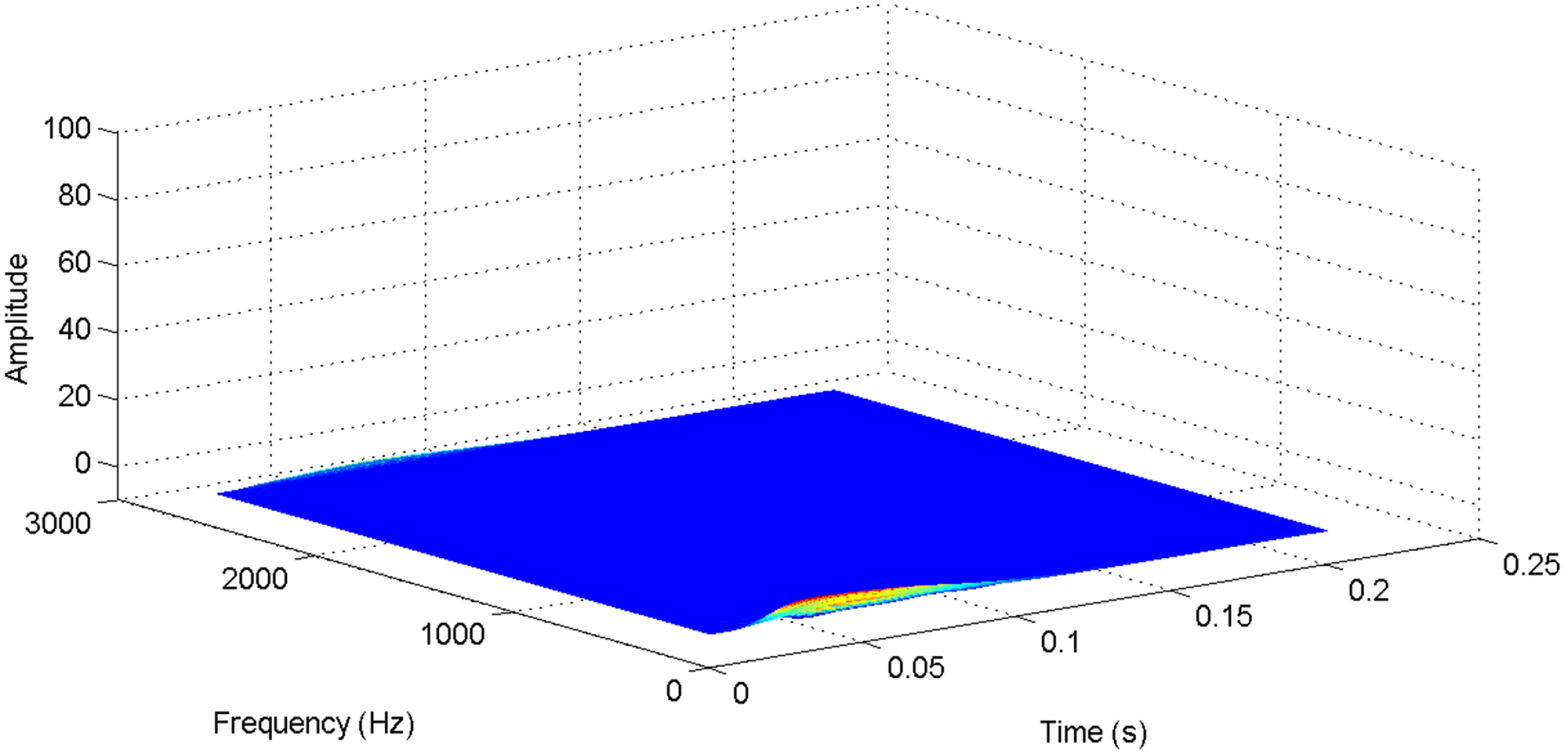
The WVD result of reactive power signal for our proposed method.

**Fig 19:**
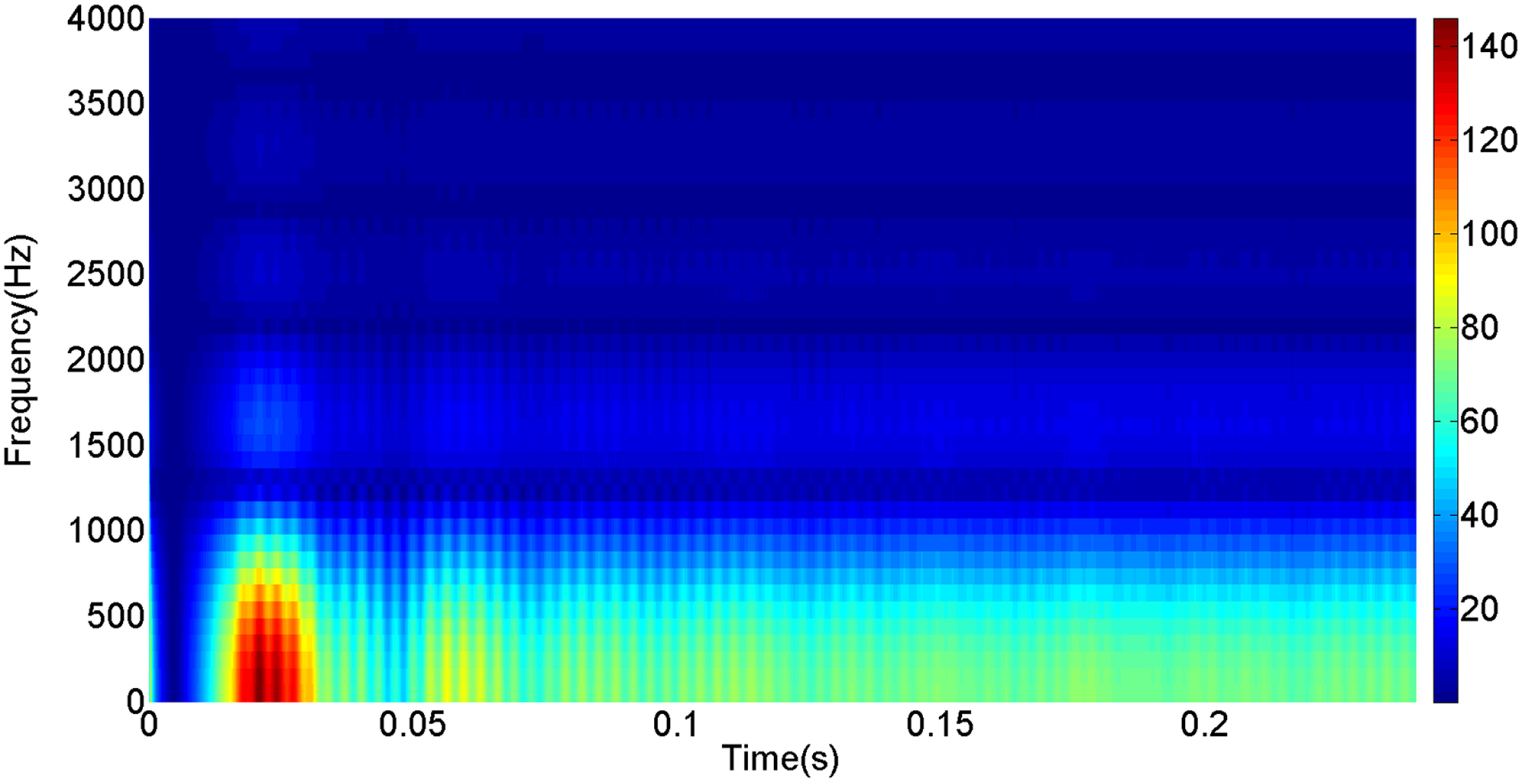
The AOK TFR result of DC current signal for PI method.

**Fig 20:**
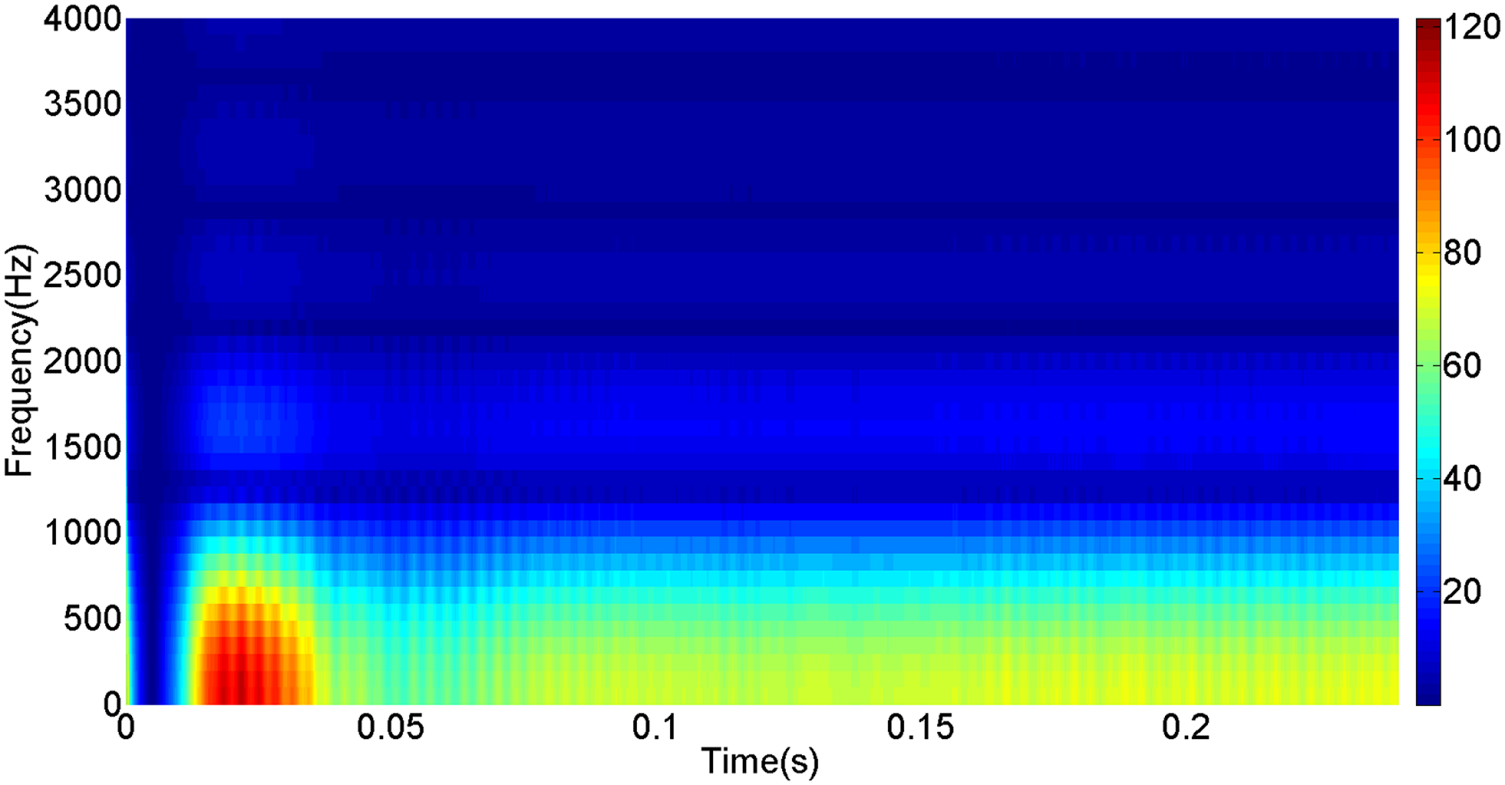
The AOK TFR result of DC current signal for our proposed method.

**Fig 21:**
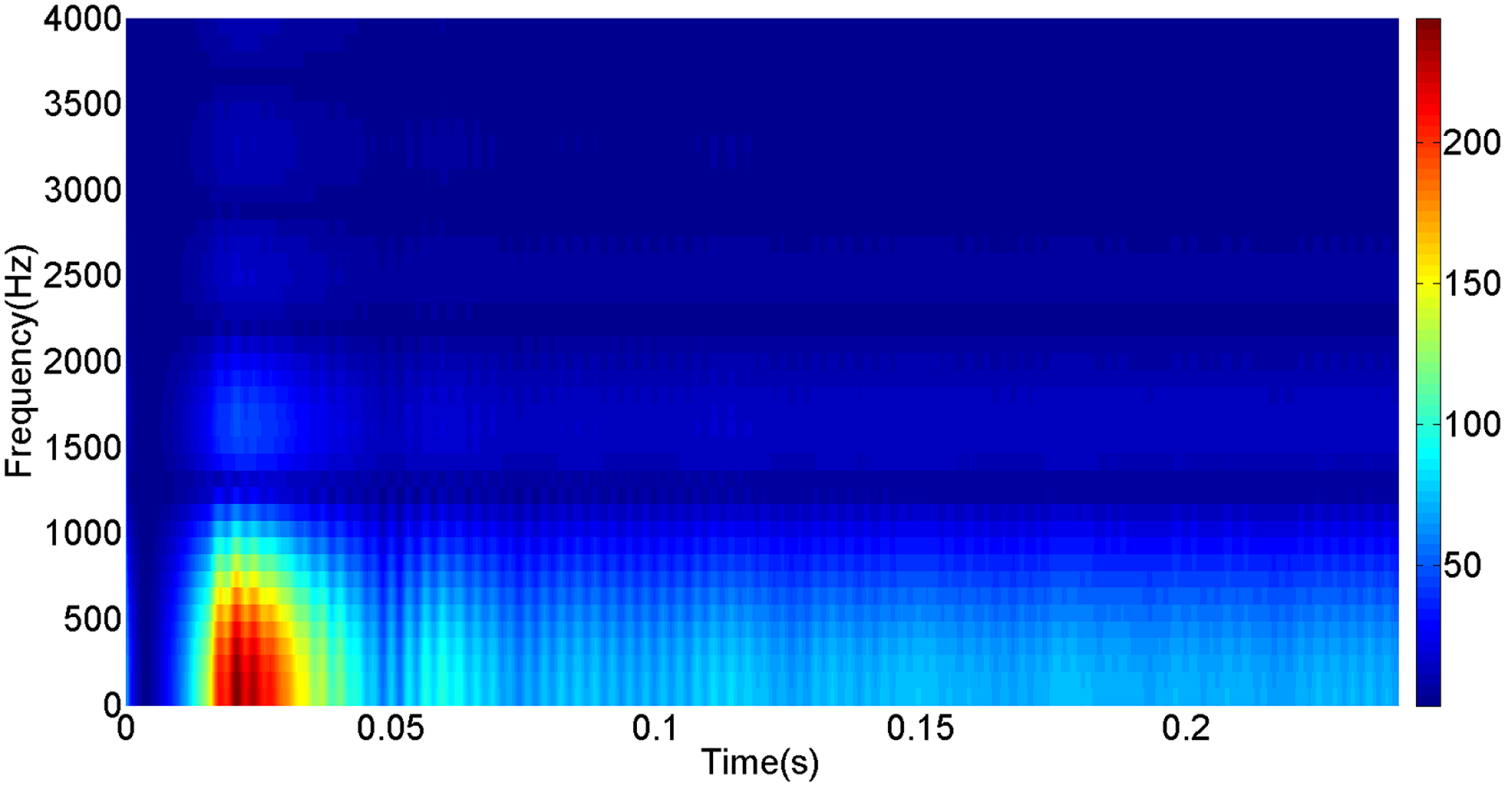
The AOK TFR result of transmitted power signal in DC side for PI method.

**Fig 22:**
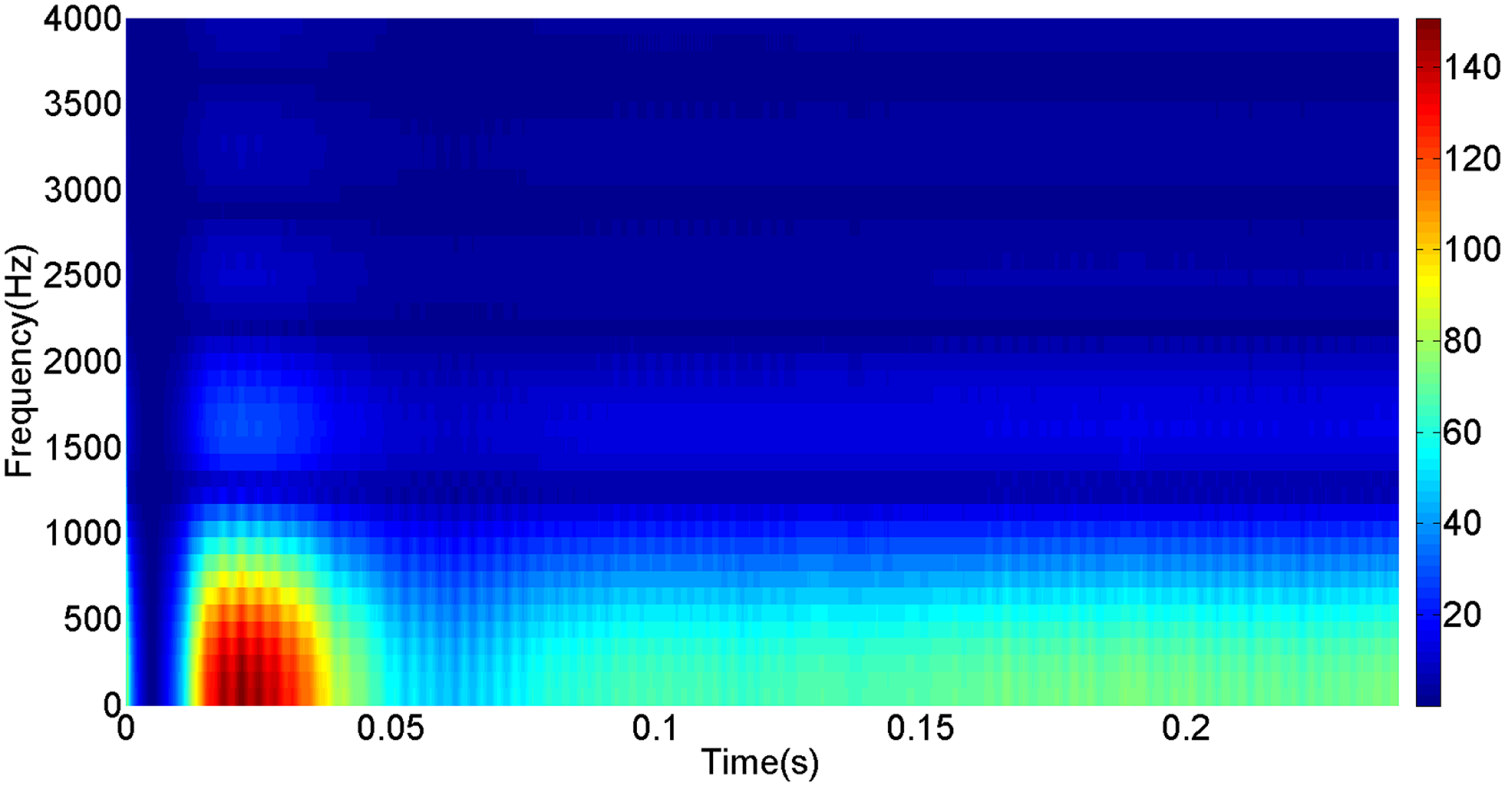
The AOK TFR result of transmitted power signal in DC side for our proposed method.

**Fig 23:**
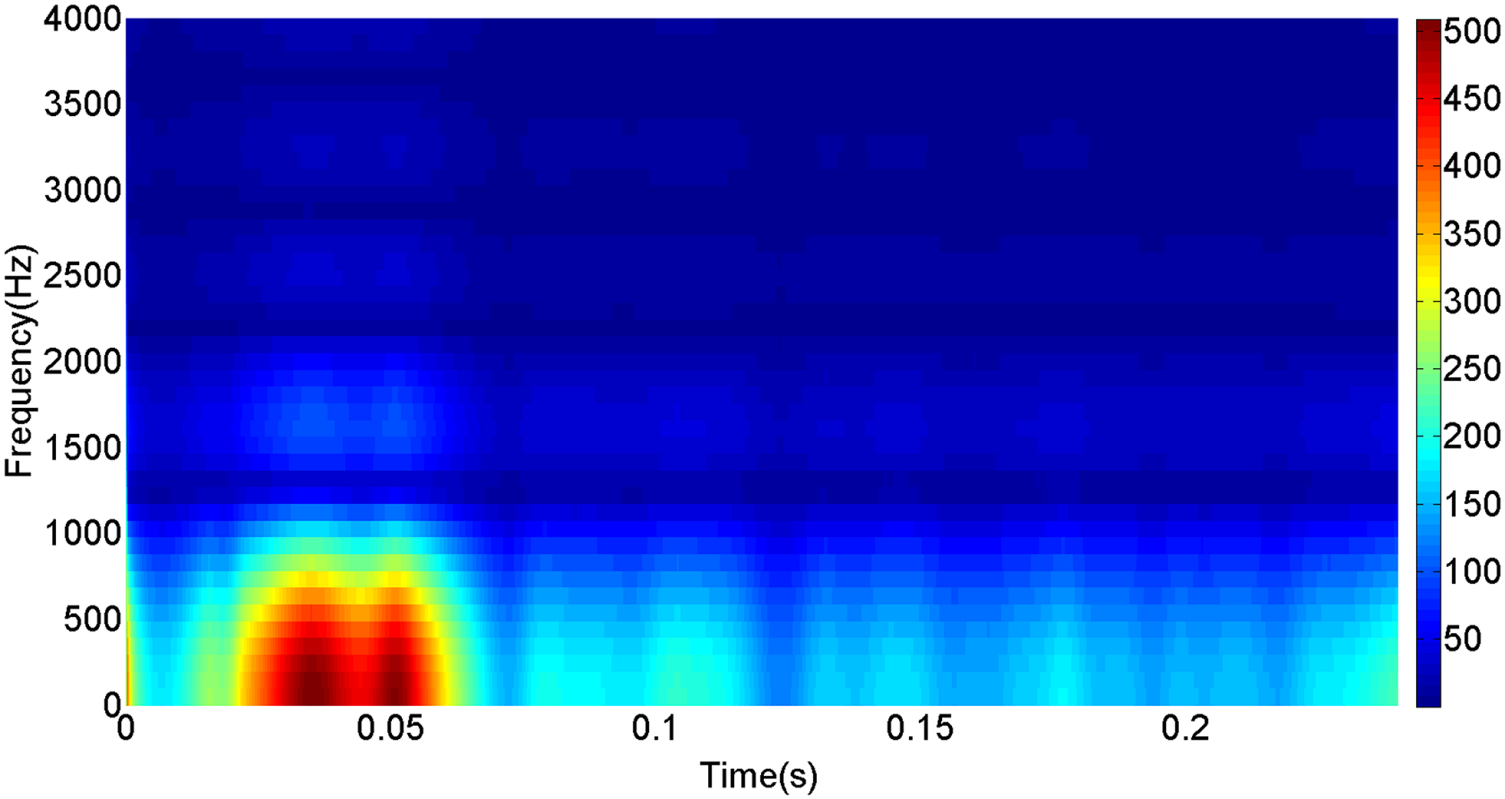
The AOK TFR result of active power signal for PI method.

**Fig 24:**
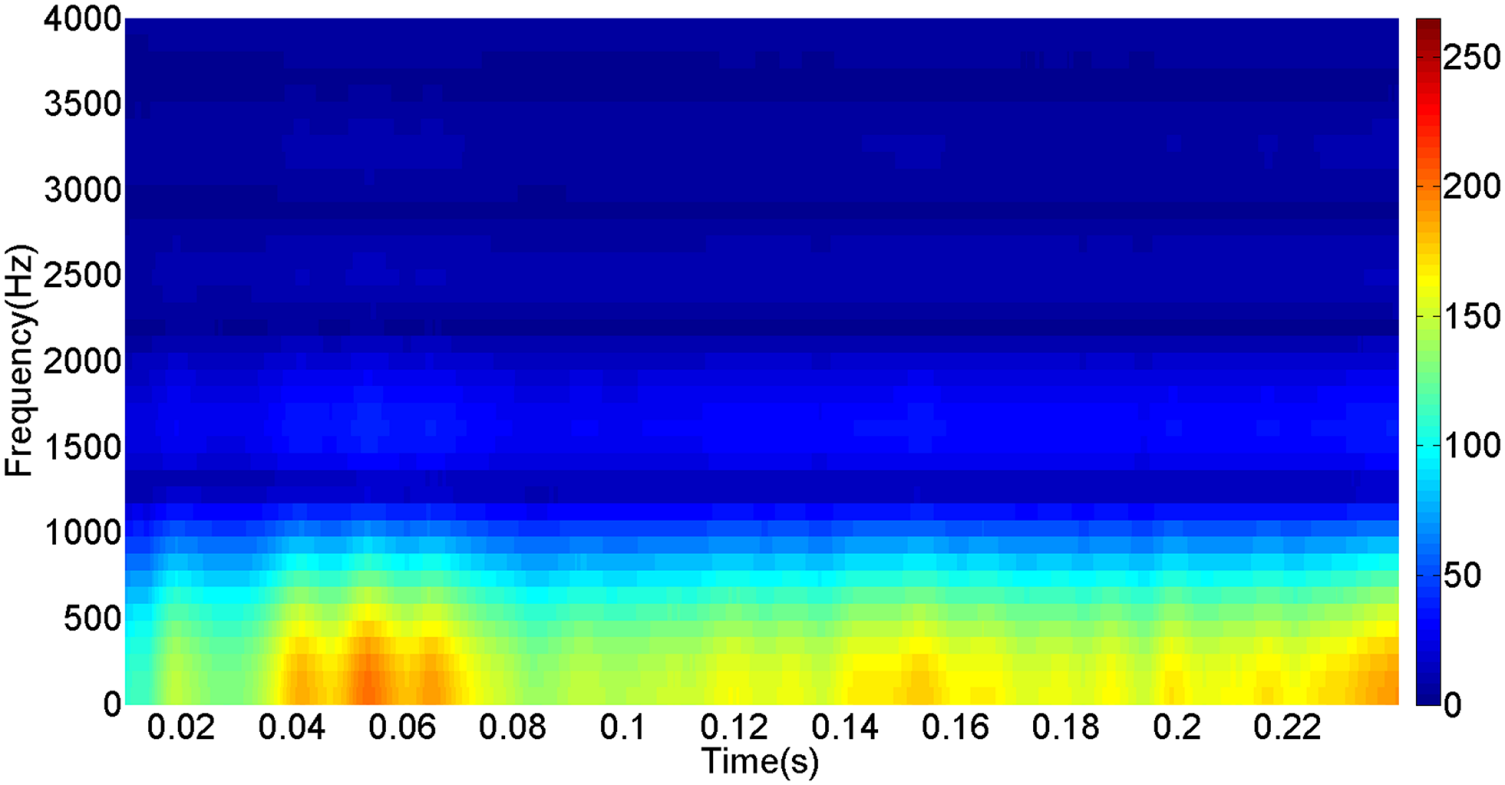
The AOK TFR result of active power signal for our proposed method.

**Fig 25:**
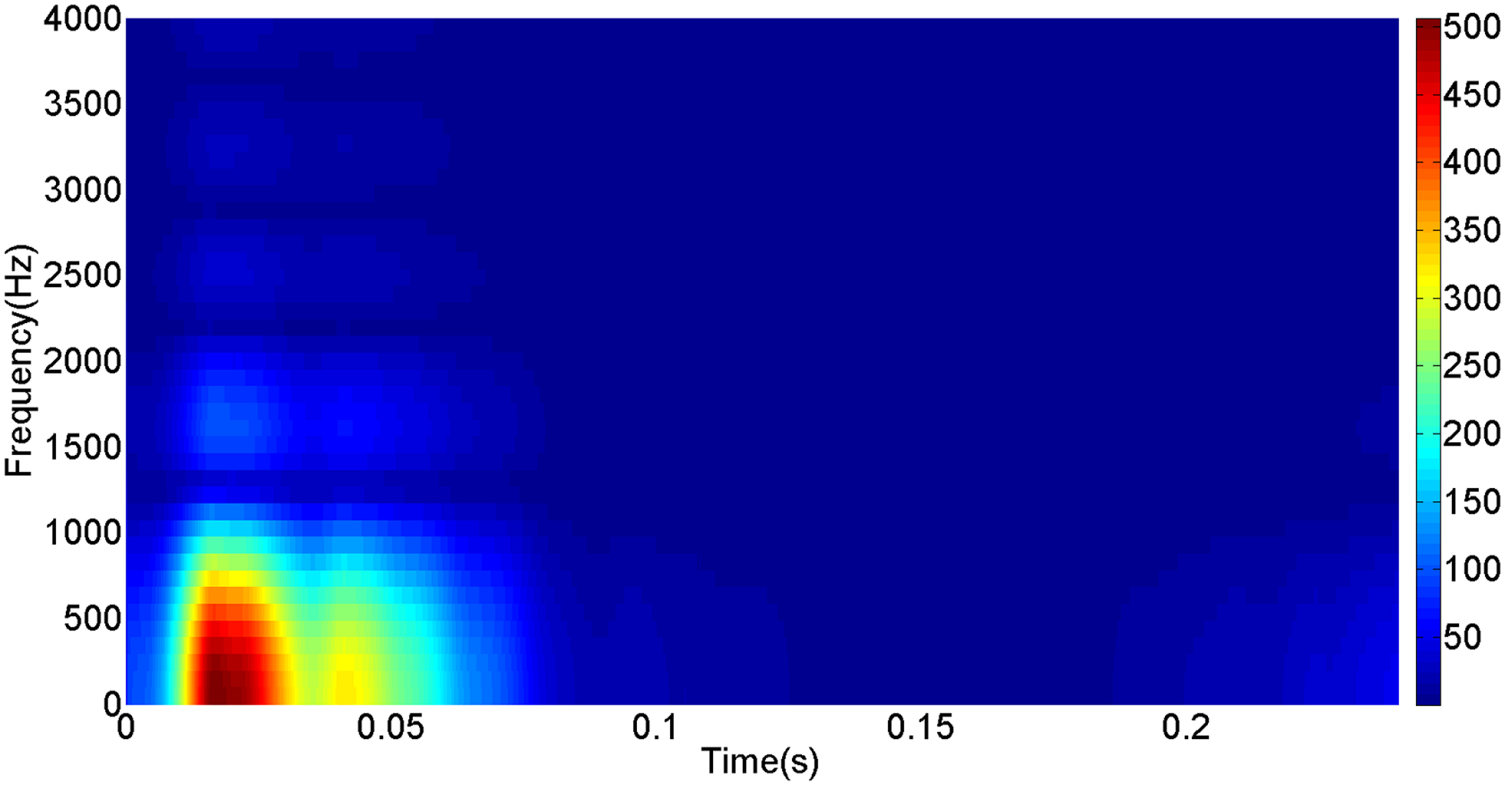
The AOK TFR result of reactive power signal for PI method.

**Fig 26:**
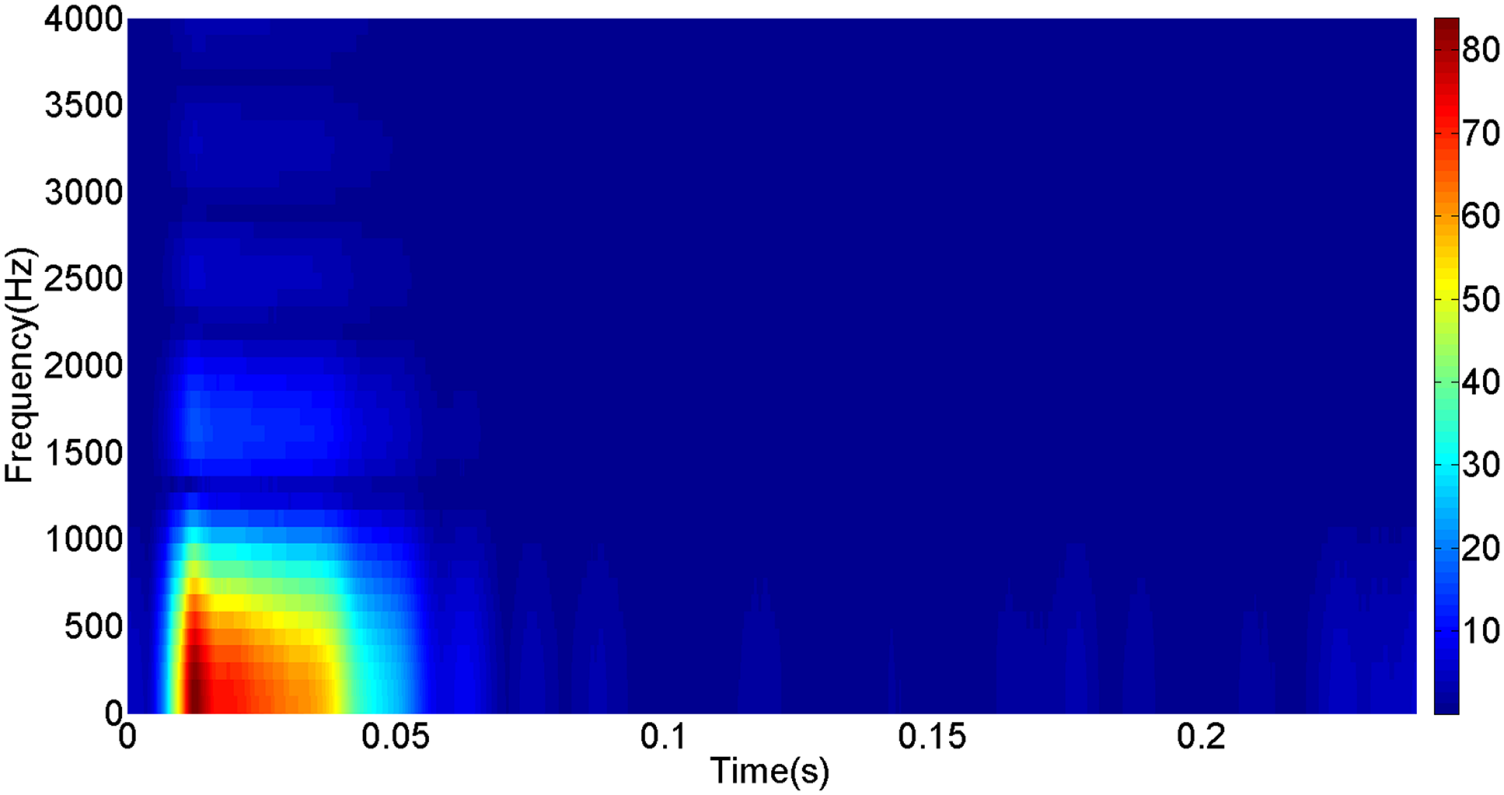
The AOK TFR result of reactive power signal for our proposed method.

More details about the WVD method and AOK TFR method see Ref. [49–50]. The AOK time frequency representation can effectively suppress the cross-term while keeping a high time-frequency concentration. From the above definitions we could know that, WVD and AOK TFR give a mapping from time domain to time-frequency domain, which means it makes analyzing signals in both time and frequency domain possible. The corresponding WVD results for our proposed method and PI method are shown in Figs 11 and 12, Figs 13 and 14, Figs 15 and 16, Figs 17 and 18, respectively. The corresponding AOK TFR results for our proposed method and PI method are shown in Figs 19 and 20, Figs 21 and 22, Figs 23 and 24, Figs 25 and 26, respectively.

Fig 3 presents the DC current wave for PI method, and Fig 4 shows the DC current wave for our proposed method. From Fig 3, we can see that, when the system starts up, the DC current overshoot comes to 0.345pu. Then it oscillates up to 0.15s. When the given DC voltage changes from 1pu to 0.7pu at 0.3s, the DC current drops to nearly 0.13pu with a small overshoot. In regard to the steady-state performance, we can see clearly that the steady-state error is large, and the waves always fluctuate. By contrast, for results of our proposed method in Fig 4, the overshoot is 0.27pu, and it is smaller than PI method. Then it quickly comes to a steady state. When the given DC voltage changes from 1pu to 0.7pu at 0.3s, the DC current changes to a new steady state faster without overshoot. From the wave, we can see that it is smoother, and the steady-state error is smaller. The steady state performance of the system has been improved. The WVD results of DC current signals for PI method and our proposed method are shown in Figs 11 and 12, respectively. And the AOK TFR results of DC current signals for PI method and our proposed method are shown in Figs 19 and 20, respectively. From the WVD results, we can see that, the maximum energy value for PI method mainly lies in 9 while for our proposed method it decrease to 6, indicating the DC current overshoot is reduced. From the AOK TFR results, we can see that, the energy value reaches to 140 for PI method, while for our proposed method it decrease to 110, indicating that the system overshoot has been reduced by using our proposed method. Moreover, after 0.05s, there exists some energy intermittent distribution for PI method, while the energy distribution becomes steady for our proposed method, demonstrating that the steady performance is improved.

Fig 5 shows the transmitted power wave in DC side for PI method, and Fig 6 presents the transmitted power wave in DC side for our proposed method. For PI method, the transmitted power in DC side has a large overshoot with the value of 0.5pu for the beginning. When the given DC voltage changes to 0.7pu at 0.3s, the transmitted power in DC side drops to 0.1pu, but the overshoot still exists together with a large steady-state error. However, for the proposed method, the overshoot becomes smaller, i.e., 0.35pu. And the steady-state error becomes smaller, indicating the control accuracy becomes higher. These results suggest that the ADRC optimized by LSSVM controller has improved the observation accuracy. The WVD results of transmitted power signals in DC side for PI method and our proposed method are shown in Figs 13 and 14 respectively. And the AOK TFR results of transmitted power signals in DC side for the two methods are shown in Figs 21 and 22, respectively. From the WVD results, we can see that, the maximum energy value for PI method mainly lies in 20 while for our proposed method it decrease and focuses on 12, indicating the transmitted power overshoot is reduced. From the AOK TFR results, we can see that, the energy value reaches to 250 for PI method, while for our proposed method it decrease to 130, which once again suggesting that the transmitted power overshoot has been obviously reduced under our proposed method. In addition, after 0.05s, there exist some energy intermittent distribution for PI method and the energy distribution for our proposed method is steady, indicating that the steady performance is improved. These time-frequency analysis results suggest that our proposed method performs much better than the traditional PI method.

Fig 7 presents the active power wave for PI method, and Fig 8 shows the active power wave for our proposed method. Fig 9 gives the reactive power wave for PI method, and Fig 10 shows the reactive power wave for our proposed method. Through comparing the two methods, we can draw a conclusion that, whether the active power or the reactive power, the starting overshoots become much lower via our proposed method and the steady-state error is much smaller. Meanwhile, the decoupling control of the active power and the reactive power has also been realized via our proposed method. The WVD results of active power signals for PI method and our proposed method are shown in Figs 15 and 16. The WVD results of reactive power signals for PI method and our proposed method are shown in Figs 17 and 18. From the above WVD results, the maximum energy value of active power reaches to nearly 70 and 10 for the two methods, respectively. And the maximum energy value of reactive power reaches to nearly 60 and 5 for the two methods, respectively. These indicate that the overshoots of the active power and reactive power have been reduced. The AOK TFR results of active power signals for PI method and our proposed method are shown in Figs 23 and 24, respectively. And the AOK TFR results of reactive power signals for PI method and our proposed method are shown in Figs 25 and 26, respectively. From the AOK TFR results, we can see that, the energy value of active power waves reaches to 500 for PI method, while for our proposed method it decreases to 180. And the energy value of reactive power waves reaches to 500 for PI method, while for our proposed method it decreases to 80, indicating that the overshoot has been effectively reduced and the better performance of our proposed method has been verified.

The nomenclature of this paper is shown in Table 2.

**Table 2:** The nomenclature.

Variable name	Variable
the three phase voltage instantaneous values of the AC power system	*u_ca_*,*u_cb_*,*u_cc_*
the three phase voltage instantaneous values of the convertor	*u_sa_*,*u_sb_*,*u_sc_*
the three phase currents	*i_a_*,*i_b_*,*i_c_*
the DC voltage	*u_dc_*
the converter transformer inductance	*L*
the equivalent resistance	R
the DC current	*I_dc_*
the load current	*I_dl_*
the input signal	*v*(*t*)
the transient process of *v*(*t*)	*v*_1_
the n-1 order differential signal	*V_n_*
the output signal of the control object	*y*(*t*)
the state variables estimated by ESO	*z*_1_….*z_n_*
the system disturbance estimated by ESO	*z*_n+1_
the errors of *v*_1_…..*v*_n_ and *z*_1_…..*z*_n_	*e*_1_….*e*_n_
the initial control signal	*u*_0_(*t*)
the final control signal	*u*(*t*)
the compensating factor	B
tracking speed factor	*r*
sample time	*T*
filter factor	*δ*
nonlinear factors	*α*_1_,*α*_2_
correction gains of output error	*β*_01_,*β*_02_
the nonlinear function	*fal*(*e*,*α*,*δ*)
the given DC voltage	$U_{DC}^{*}$
the given d-axis component	$i_{d}^{*}$
the input data	*x_k_*
the output data	*y_k_*
the kernel space mapping function	*φ*():*R^n^*→*R^m^*
the weight vector	*w*ϵ*R^m^*
the error vector	*e_k_*ϵ*R*
the offset	*C*
the Lagrange factor	*α_k_*ϵ*R*
the kernel function	k(·,·)
a kernel function for generating AOK TFR	Φ(*τ*,*υ*)
a window signal	*A*(*t*,*τ*,*υ*)
the time-delay	*τ*
the frequency shift	*υ*

## Conclusions

We have investigated and proposed an ADRC optimized by LSSVM control method, which allows improving the rectifier side performances in VSC-HVDC system. We construct the mathematical model of the proposed method used in DC voltage loop. In addition, our simulations and time-frequency representation results have verified the effectiveness of the proposed method. As further works, we will develop an auto self-adapting algorithm to adjust ADRC parameters online and establish an experimental platform to carry out experiments. In summary, we have drawn the following conclusions.

1. The steady state performance of the system has been improved.
2. The quick dynamic response has been kept.
3. The decoupling control of the active power and the reactive power has been realized.
4. The time-frequency representation (WVD and AOK TFR) methods can be successfully used to analyze the VSC-HVDC system signals, which open up a new application field.
